# Comprehensive Binary Interaction Mapping of SH2 Domains via Fluorescence Polarization Reveals Novel Functional Diversification of ErbB Receptors

**DOI:** 10.1371/journal.pone.0044471

**Published:** 2012-09-04

**Authors:** Ronald J. Hause, Kin K. Leung, John L. Barkinge, Mark F. Ciaccio, Chih-pin Chuu, Richard B. Jones

**Affiliations:** 1 The Committee on Genetics, Genomics, and Systems Biology, The Ben May Department for Cancer Research and the Institute for Genomics and Systems Biology, The Gwen and Jules Knapp Center for Biomedical Discovery, The University of Chicago, Chicago, Illinois, United States of America; 2 The Committee on Cancer Biology, The Ben May Department for Cancer Research and the Institute for Genomics and Systems Biology, The Gwen and Jules Knapp Center for Biomedical Discovery, The University of Chicago, Chicago, Illinois, United States of America; 3 The Committee on Cellular and Molecular Physiology, The Ben May Department for Cancer Research and the Institute for Genomics and Systems Biology, The Gwen and Jules Knapp Center for Biomedical Discovery, The University of Chicago, Chicago, Illinois, United States of America; 4 Institute of Cellular and System Medicine, and Translational Center for Glandular Malignancies, National Health Research Institutes, Miaoli, Taiwan; University of Edinburgh, United Kingdom

## Abstract

First-generation interaction maps of Src homology 2 (SH2) domains with receptor tyrosine kinase (RTK) phosphosites have previously been generated using protein microarray (PM) technologies. Here, we developed a large-scale fluorescence polarization (FP) methodology that was able to characterize interactions between SH2 domains and ErbB receptor phosphosites with higher fidelity and sensitivity than was previously achieved with PMs. We used the FP assay to query the interaction of synthetic phosphopeptides corresponding to 89 ErbB receptor intracellular tyrosine sites against 93 human SH2 domains and 2 phosphotyrosine binding (PTB) domains. From 358,944 polarization measurements, the affinities for 1,405 unique biological interactions were determined, 83% of which are novel. In contrast to data from previous reports, our analyses suggested that ErbB2 was not more promiscuous than the other ErbB receptors. Our results showed that each receptor displays unique preferences in the affinity and location of recruited SH2 domains that may contribute to differences in downstream signaling potential. ErbB1 was enriched versus the other receptors for recruitment of domains from RAS GEFs whereas ErbB2 was enriched for recruitment of domains from tyrosine and phosphatidyl inositol phosphatases. ErbB3, the kinase inactive ErbB receptor family member, was predictably enriched for recruitment of domains from phosphatidyl inositol kinases and surprisingly, was enriched for recruitment of domains from tyrosine kinases, cytoskeletal regulatory proteins, and RHO GEFs but depleted for recruitment of domains from phosphatidyl inositol phosphatases. Many novel interactions were also observed with phosphopeptides corresponding to ErbB receptor tyrosines not previously reported to be phosphorylated by mass spectrometry, suggesting the existence of many biologically relevant RTK sites that may be phosphorylated but below the detection threshold of standard mass spectrometry procedures. This dataset represents a rich source of testable hypotheses regarding the biological mechanisms of ErbB receptors.

## Introduction

The human ErbB family [1] comprises four receptor tyrosine kinases (RTKs): EGFR/ErbB1 [2]; ErbB2/HER2 [3]; ErbB3 [4], [5]; and ErbB4 [6] which are activated in response to extracellular growth factors. ErbB2 has no known ligands [7], but is the preferred hetero-dimerization partner of other ligand-bound members of the ErbB family [8]. ErbB3 can recognize growth factor ligands leading to activation of the tyrosine kinase activity of its hetero-dimerization partners but contains much lower intrinsic kinase activity [9] than the other receptor family members. Following growth factor binding, activated receptor dimers are auto-phosphorylated in *trans* on intracellular tyrosines. Signaling proteins containing Src homology 2 (SH2) [10]–[12] and phosphotyrosine binding (PTB) domains [13] physically interact with a subset of these phosphorylated tyrosines. Proteins containing these domains then elicit downstream molecular functions that induce changes in cell behavior including proliferation, migration, cytoskeletal rearrangement, and enhanced survival [14], [15]. The ErbB RTKs play important roles in both normal and aberrant cell processes including cancer [16], [17]. However, many unanswered questions remain regarding the mechanisms of ErbB receptor signal propagation. Although most autophosphorylated tyrosines on the receptors have been mapped and reported in multiple literature sources, heterophosphorylation sites–those phosphorylation events requiring the activity of recruited kinases–have rarely been reported [18]–[21]. The possibility therefore exists that many physiologically relevant phosphorylation events occur locally in the cell that might escape characterization via mass spectrometry approaches and that may represent potentially important and unappreciated sources of biological activity. Orthogonal approaches aimed at identifying biologically relevant phosphorylation sites are critical for the characterization of novel ErbB receptor functions that occur through phosphorylation events of low stoichiometry or that are mediated through weak and dynamic protein interactions.

Previous studies have characterized the regulatory role of a small subset of SH2 domains, including those from c-Src (SRC) [22]–[24] and PLCγ1 (PLCG1) [25]. However, we still lack a systematic understanding of how most SH2 domains regulate the cellular function of their host proteins. Surface plasmon resonance (SPR) [26], [27] and isothermal titration calorimetry (ITC) methods [28]–[30] have been used to query the interaction affinities between several SH2 domains and several phosphorylated peptide substrates. Because of cost, time, and labor, SPR and ITC have not been adapted for systematically probing comprehensive interaction matrices of SH2 domains with potential RTK phosphorylation sites.

Generalized binding preferences for most SH2 domains have previously been defined through screening approaches using degenerate peptide libraries [31], [32]. In a more targeted fashion, about 100 peptides derived from FGF, IGF, and insulin receptors have also been similarly screened [33]. Protein microarrays (PMs) have been used to semi-quantitatively interrogate the binding potential of matrices of SH2 domains and dozens of peptides derived from phosphotyrosine sites of the ErbB [34] and other RTK families [35], [36]. One systems-level conclusion from these studies was that ErbB1 and ErbB2 recruited a dramatically higher number of unique SH2 domains versus ErbB3 and ErbB4 as the affinity threshold was reduced to include weaker interactions. Overexpression of ErbB1 or ErbB2 (rather than of ErbB3 or ErbB4) by cancerous cells was therefore predicted to result in the recruitment and activation of a larger subset of SH2 domain-containing signaling molecules [34], [35]. The greater SH2 recruitment capacity of ErbB1 and ErbB2 was hypothesized to confer greater oncogenic potential to cancer cells than ErbB3 and ErbB4 given that many SH2 domains are contained in proteins that facilitate mitogenesis, cell survival, and cell motility [34]. However, those studies queried phosphopeptide interactions corresponding to a small subset of all intracellular ErbB tyrosines.

Previous phosphopeptide query sets have been heavily biased towards ErbB receptor auto-phosphorylation sites, which are likely to represent the sites of highest phosphorylation stoichiometry following ErbB activation and have been most often reported by mass spectrometry based literature reports. Additionally, PMs were not able to accurately or reproducibly quantify interactions with midpoint dissociation constants (K_D_s) weaker than 2 µM because of technical limitations related to concentration-dependent aggregation of rhodamine-labeled peptides [34]. Nevertheless, physiologically relevant SH2 domain-mediated interactions have been reported with K_D_s much weaker than 2 µM. For example, the interaction of the c-Src SH2 domain with physiological PDGFR phosphosites has been estimated at approximately 4 µM [37] through peptide competition analysis. Such exemplary studies indicate that many physiologically relevant interactions may exist between SH2 domains and cellular phosphotyrosine sites that have not been previously captured because of technical limitations.

Fluorescence polarization (FP) has previously been used to characterize relatively weak protein interactions including the binding of fluorescent peptides to the c-Src SH2 domain [38] and to a large number of PDZ domains [39]. FP was also used as a high-confidence assay for cross-validating subsets of interactions determined by PMs [40] because of the high accuracy and wide dynamic range of data obtained by the method. We therefore implemented a high-throughput version of the FP method to interrogate the interaction potential of ErbB phosphopeptides with SH2 domains. Our analysis recaptured many previously reported interactions and nearly 1200 unreported ones and indicated that many interactions previously identified by PMs may potentially be false positives. Moreover, our FP-derived dataset, which employed a more comprehensive set of ErbB phosphopeptides, suggested a substantially different systems-level recruitment potential of the ErbB RTKs than has been suggested from previous studies.

## Materials and Methods

A more comprehensive description of methods is provided in the Supplementary Methods file.

### SH2 and PTB Domain Proteins

The cloning of 109 SH2 and 44 PTB domains in the human genome is previously described [34]. In the current study, 93 SH2 and 2 PTB domain-containing constructs (Figure S1, Table S1) were selected that met each of the following criteria: 1) fraction of monomeric protein observed in previous study following expression and purification ≥50% by size exclusion chromatography; 2) previous evidence of functionality by PM as evidenced by interaction with one or more phosphopeptides with an apparent midpoint binding constant K_D_≤1 µM. Where multiple SH2 domains were contained in a single gene, the tandem protein was included in our analysis with all internal amino acids linking the domains even if the percentage of monomeric tandem SH2 domains was less than 50%.

### Peptide Synthesis and Purification

Peptides were synthesized on a Symphony® 12 channel multiplex peptide synthesizer (Protein Technologies, Inc., Tucson, AZ) in either N,N-dimethylformamide (DMF) (Sigma-Aldrich, St. Louis, MO) or N-Methyl-2-Pyrrolidone (NMP) (Advanced ChemTech, Louisville, KY) on the solid phase at a 50 µmol scale using standard Fmoc chemistry. Standard amino acids were coupled twice for 30 minutes at five-fold molar excess with the exception of leucine, valine, isoleucine, threonine and tryptophan, which were coupled three times for 45 minutes; phosphotyrosine was coupled twice for three hours; standard amino acids were coupled twice for one hour following phosphotyrosine addition. In most cases, amino acids were activated in situ with 0.9 equivalents of 2-(1H-benzotriazole-1-yl)-1,1,3,3-tetramethyluronium hexafluorophosphate (HBTU), one equivalent of 1-Hydroxybenzotriazole (HOBT), and two equivalents of N,N-diisopropylethylamine (DIPEA) (Advanced ChemTech, Louisville, KY) and coupled at room temperature. Phosphotyrosine was pre-activated separately for 30 minutes prior to coupling. Either standard polystyrene Wang resin (0.6 mmol/g) charged with Fmoc-Asp (OBut) (Advanced ChemTech, Louisville, KY) or CLEAR-Acid resin (0.4 mmol/g) charged with Fmoc-Asp (OtBu) (Peptides International, Louisville, KY) was used to initiate peptide chains. Following synthesis but before cleavage, peptides were N-terminally labeled twice for four hours with two equivalents of rhodamine WT (Abbey Color, Philadelphia, PA) activated with equimolar amounts of HBTU, HOBT, and three equivalents of DIPEA. 5 mM DTT was added to all cysteine-containing peptides prior to peptide analysis and purification.

### Fluorescence Polarization (FP) Saturation Binding Assay

**Figure 1:**
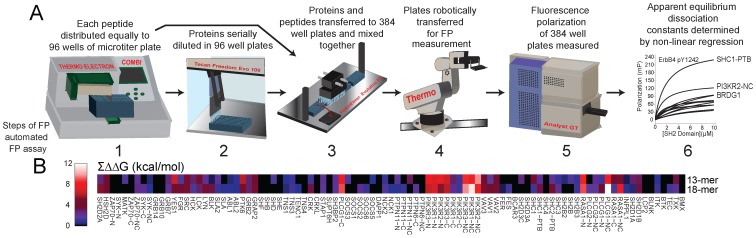
Automated high-throughput fluorescence polarization (FP) procedural schematic. (**A**) (**1**) Following synthesis and mass-directed purification, 10 µL of phospho-peptide (10 nM) labeled on the N-terminus with rhodamine is distributed equally to each well of a 96-well plate. (**2**) After expression and purification, 8 SH2 domains at an original concentration of 20 µM are serially diluted 11 times in two-fold increments into 96-well plates. (**3**) Proteins from 96-well plates are added in four pipetting steps to a single 384-well plate. To the same plate, 12 different concentrations of eight different SH2 domains are added to each quadrant of the 384-well plate. In total, 32 unique SH2 domains at 12 different concentrations are mixed with a single peptide. (**4**) Following an incubation period of 20 min, 384-well plates of SH2 domains and peptides are delivered to the Analyst GT (**5**) for measurement of FP. Data from these measurements are used for determining apparent dissociation constants (**6**). (**B**) Comparison of binding data using 13-mer- and 18-mer peptides. Heat maps depict summations of relative binding free energies (ΔΔG, kcal/mol) as a function of apparent K_D_s of SH2 and PTB domains interacting with indicated peptides (see equation 1 in text). ΔΔG summations are color coded by binding strength.

(See Figure 1A) Rhodamine-labeled peptides were dissolved in assay buffer (20 mM HEPES, 100 mM KCl, 0.1% Tween-20, 5 mM DTT, pH 7.8), diluted to a concentration of 20 nM, and plated into black 384-well plates (Corning, Corning, NY) at 5 µL/well using a Perkin Elmer EP3 pipetting workstation (V&P Scientific, Inc., San Diego, CA). SH2, PTB, and thioredoxin control proteins were plated into the first column of 2 mL 96-well plates (Corning) at a concentration of 20 µM and serially diluted 1∶2 into freezing buffer (300 mM NaCl, 50 mM Na2PO4, pH 8, 20% glycerol v/v) eleven times using a Tecan Freedom EVO 100 workstation (Tecan, Männedorf, Switzerland). For the interaction assay, 5 µL of each of the twelve protein concentrations was added to the peptide-containing 384-well plates via the EP3 resulting in a protein dilution range of 0.002–10 µM and a final volume of 10 µL; peptide concentration was fixed at 10 nM. Peptide/protein samples were incubated for 20 minutes at room temperature in the dark and fluorescence polarization (FP) was measured in an Analyst GT multimode reader (Molecular Devices, Sunnyvale, CA) with excitation and emission wavelengths of 530 nm and 555 nm, respectively. Experimental values were output as millipolarization (mP) units and imported into MATLAB (The MathWorks, Inc., Natick, MA) in which equation (1) was used to determine dissociation constants (K_D_s) for each protein/peptide pairing by least squares nonlinear regression. 
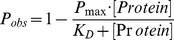
(1)


### Z’ Factor Determination

To evaluate the suitability of a subset of binary interaction partners for examination in competitive binding assays, we determined *Z’* factors for each putative interaction detected by FP. The Z’ factor is defined in equation (2) as:
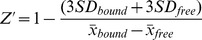
(2)in which 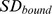 and 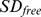 are the standard deviations and 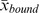 and 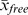 are the means of the polarization signals (mP) for bound and free peptide, respectively. For positive controls of the bound state, we incubated 10 nM of each rhodamine-labeled peptide with a concentration of protein equivalent to four times the K_D_ of that interaction. For negative controls of the free state, we incubated identical concentrations of each labeled peptide with a thioredoxin control at the same concentration as the positive control protein. All Z’ factor calculations were performed in R (http://www.R-project.org).

### FP Competition Assay

Unlabeled phosphopeptides were synthesized with some modifications to the previously described protocol. Final fluorescent peptide and protein concentrations were kept constant at 10 nM and four times the calculated K_D_ of the interaction (based on the FP saturation binding assay), respectively. Unlabeled competitor peptides were prepared at 11 serial 1∶3 dilutions in assay buffer; one control with no unlabeled competitor peptides was also included with each experimental set. The fluorescent peptide, protein, and unlabeled peptide mixtures were allowed to incubate for a minimum of 20 minutes in 384-well plates and polarization signals were measured using the Analyst GT multimode reader as previously described above. Inhibition curves were constructed by four-parameter log-logistic regression using the “drc” package [41] in R with the parameters for maximum and minimum fluorescence fixed according to *Z*’ factor positive and negative control values. IC_50_ values were estimated from these fits under a one-site competition binding model and K_i_ calculated according to the equation developed by [42].

### Protein Microarrays

300 pL of purified recombinant proteins and a thioredoxin tag control were spotted at a concentration of ∼40 µM in duplicate onto aldehyde-coated microscope slides (Erie Scientific Company, Portsmouth, NH) using a Nano-Plotter (NP) 2.1/E noncontact microarrayer (GeSiM, Munich, Germany). 16 identical microarrays per slide were printed in a 2×8 (column by row) format with distances of 9 mm between individual arrays; each array consisted of a 14×14 configuration of spots 400 µm apart. Following printing at a constant humidity level of 50%, slides were desiccated overnight and stored at −80°C. For peptide probing, array slides were transferred to 96-well microplates (TeleChem International, Inc., Sunnyvale, CA) and sandwiched with silicone gaskets. Prior to peptide addition, aldehyde sites were quenched with buffer B (20 mM HEPES, 100 mM KCl, 0.1% Tween-20, 5 mM DTT, pH 7.8) containing 1% bovine serum albumin (BSA) (w/v) for one hour at room temperature; moderate agitation was applied in a plate shaker (IKA, Germany) to displace glycerol from the surface area of protein sample spots. After washing the arrays briefly with buffer B alone, a titration series of rhodamine-labeled peptides (whose concentration was determined by rhodamine emission at 555 nm) dissolved in buffer B was added to the PMs and incubated in the dark for one hour at room temperature with gentle agitation. The arrays were subsequently washed once with 300 µL of buffer B and once with 300 µL of ddH_2_O. Residual water was removed by centrifugation; slides were stored under desiccation in the dark until analysis.

### Surface Plasmon Resonance

All SPR experiments were performed using Biacore 3000 (GE HealthCare, Piscataway, NJ) instrumentation. 6X His-tagged SH2 domain proteins were immobilized on a nitriloacetic acid (NTA) sensor chip (GE HealthCare, Piscataway, NJ) charged with Ni^2+^ ions. Dye-free ErbB family peptides were applied to the sensor chip in a single flow cell in solution phase (while the other flow cell was used as a reference control) and binding events were measured under equilibrium conditions. FP false positive 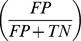 (FPR), false discovery 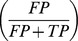 (FDR), and false negative 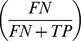 (FNR) rates were calculated by defining true positives (TP) as interactions detected by both FP and SPR, true negatives (TN) as interactions detected by neither FP nor SPR; false positives as interactions identified by FP but not validated by SPR; and false negatives (FN) as interactions missed by FP but detected by SPR. Additional details for all methods are provided in the Supplementary Methods.

## Results

### Development of High-throughput Fluorescence Polarization Assay

To scale up the number of interaction measurements while minimizing variation introduced by human intervention, we utilized an automated robotic platform (Figure 1A; see experimental procedures and Supplementary Methods) to transfer, incubate, and measure the fluorescence polarization (FP) induced from interactions of a titration series of SH2 domains with synthetic rhodamine-labeled ErbB phosphopeptides. Experimental values were output as millipolarization (mP) units and equation (1) was used to fit apparent midpoint dissociation constants (K_D_s) for each protein/peptide pair using non-linear regression (see experimental procedures).

In our previous study [34], we synthesized phosphopeptides containing nine amino acids N-terminal to the phosphotyrosine in order to ensure that sufficient peptide sequence was present for recognition by many previously untested SH2 domains. In order to maximize the polarization signals generated from interactions of peptides with SH2 domains while maintaining sufficient N-terminal length for maximal interaction selectivity, we tested the relative interaction strengths and FP values of a subset of twenty ErbB peptides containing either four or nine N-terminal amino acids with the entire set of 93 domains that were previously shown to be functional and monomeric [34] (Figure S1, Table S1, Table S2). A global comparison of interaction affinities determined by both peptide versions revealed absolute binding free energies that were significantly correlated (*P* = 2.80×10^−14^) and not significantly different (Figure 1B; Figure S2A; Wilcoxon rank sum test *P*>0.05) for assayed protein-peptide interactions. However, 13-mer peptides generated significantly higher maximum polarization (MaxP) levels than 18-mer peptides (Figures S2B and S2C; Wilcoxon rank sum test *P* = 9.28×10^−4^). Because increased maximal polarization amplitudes would likely enable higher sensitivity and more robust determination of interaction affinities, only 13-mers were synthesized for the remaining 61 peptides.

### Quantitative Analysis of ErbB Family Recruitment using Automated FP Assay

For this interaction analysis, we synthesized and queried the interaction of phosphopeptides corresponding to 89 of 92 ErbB family intracellular tyrosines with 93 SH2 domains. Because nine peptide sequences share 100% identity between the receptors in the 4 to 7 amino acids C-terminal to the phosphorylated tyrosine, our peptide set consisted of 81 phosphopeptides corresponding to the 89 unique tyrosine containing sequences (Table S2). This peptide set represents a 50% increase over the three PM studies performed to date. We were therefore able to obtain a more systematic and unbiased view of the relative SH2 domain recruitment potential of each receptor. Although most ErbB receptor auto-phosphorylation sites have been detected frequently by mass spectrometry because of their high relative modification stoichiometry, non-auto-phosphorylation sites have been reported less frequently in the literature (Table S3). We reasoned that many physiologically relevant and uncharacterized ErbB tyrosines of low phosphorylation stoichiometry may exist and that our comprehensive interaction analysis might implicate their importance in mediating physiological interactions. We tested the majority of interactions (7099/8160 (87.0%); Figure S3) three or more times and reported standard error for all interactions that were detected in at least three FP runs (Table S4). We also reported the theoretical maximum polarization values displayed by each interaction (Table S4, Figure S4), which is the predicted polarization value of the peptide when titrated at infinite protein concentration. Analysis of this polarization data may be useful for inferring how SH2 proteins bind to different peptide sequences. Larger proteins and proteins binding closer to the N-terminal rhodamine tag of a given peptide would be expected to induce higher maximal polarization (the calculated maximal polarization value at infinite protein concentration) than proteins binding farther from the tag. In addition to phosphopeptides, we queried a representative set of 27 non-phosphopeptides against the entire domain set. Only five of 2565 potential interactions (0.2%) resulted in a detected interaction from this set (Table S5). Therefore, the remaining phosphopeptides were not assayed for interaction in non-phosphorylated form.

**Figure 2:**
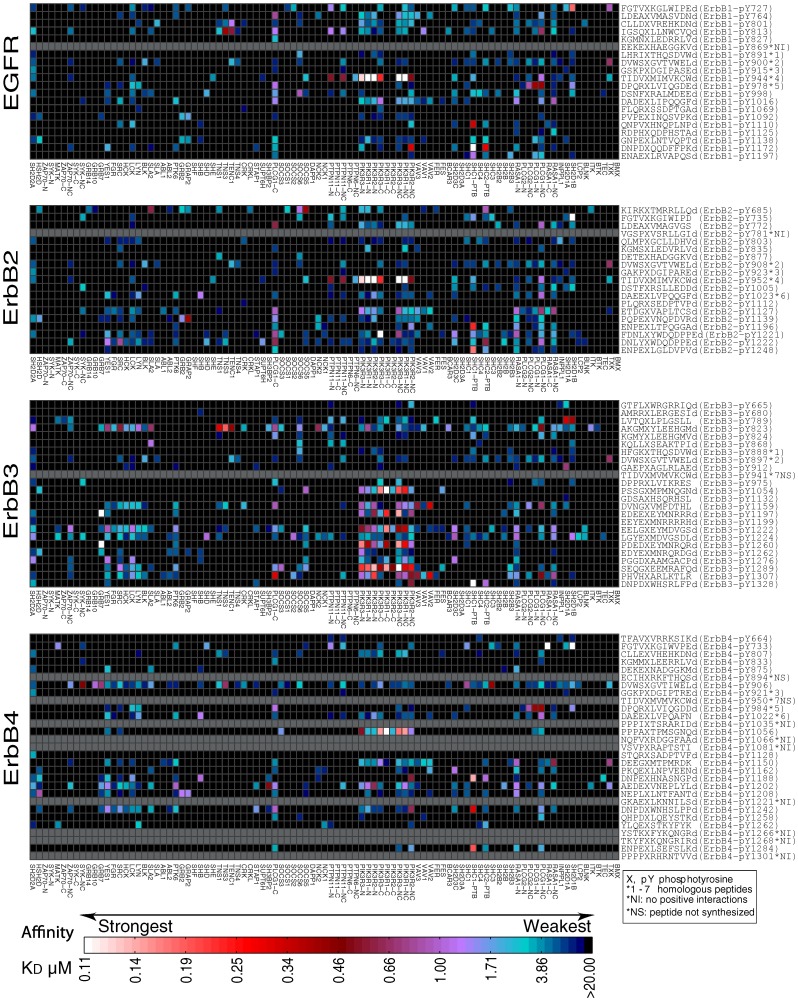
Comprehensive SH2 domain recruitment potential of the ErbB family as determined by high-throughput fluorescence polarization (HT-FP). Color-coded heat maps represent apparent dissociation constants (K_D_s) for FP interactions between SH2/PTB domains and phosphopeptides representing all potential ErbB1, ErbB2, ErbB3, and ErbB4 phosphotyrosine sites; black boxes indicate interactions that are too weak to be detected by the assay. Homologous ErbB peptides with identical amino acid residues from +1 to the +4 position relative to the phosphotyrosine (X) are indicated with an asterisk followed by the number (in order of occurrence) of the homologous receptor. Sequences of peptides used are indicated for each homologous receptor site, in which a small “d” denotes the pre-charged aspartic acid (Asp) residue on the peptide synthesis resin and not a naturally occurring Asp. NS refers to peptides that were unable to be synthesized, while NI refers to synthesized peptides that produced no positive hits in the study; therefore we cannot confirm nor deny interactions at these sites with our assay. Rows of the heatmaps for these peptides are grayed out to indicate that our FP assay could neither confirm nor deny positive or negative interactions from these peptides.

The ability of the FP method to detect weaker interactions than PMs, coupled with a more comprehensive peptide query set, revealed a much larger interaction set than has previously been described (Figure 2, Figure S5, Table S4, and Table S6). In total, our analysis with both short and long form peptides captured 1529 interactions. After filtering for redundant long and short form peptides, 1395 unique peptide-protein interactions remained. Finally, mapping the six peptides that share homology between the receptors back to their unique ErbB receptor phosphosites and extrapolating the technical interaction data to these additional phosphosites for biological inference, our analysis revealed 1405 unique biological interactions, 1169 (83.2%) of which were not described by previous PM studies. The median K_D_ of these detected interactions was 4.84 µM, an affinity much lower than the threshold filter of 2 µM used previously for PMs.

### Cross-validation of Fluorescence Polarization Assay with Surface Plasmon Resonance and Data from the Literature

To further assess the accuracy of interactions quantified by the automated FP assay, the surface plasmon resonance assay (SPR) was used to cross-validate a subset of FP interaction positive and negative hits. Because of the lower throughput nature of the SPR assay, a matrix of 63 interactions consisting of 12 peptides and seven proteins was randomly selected for this analysis (Table S7). The SPR assay shares many attributes of the FP and the PM method. It is similar to the PM assay in that proteins are immobilized onto a solid support and measured for interaction via either examination of surface plasmon resonance or fluorescence intensity, respectively, following a titration series with different concentrations of peptides. The SPR assay is also similar to the FP assay in that the procedures are automated and interaction signals are measured in real time during the incubation of proteins and peptides. By contrast, interaction strengths inferred from the PM assay are accomplished by fitting fluorescence intensities of spots on a microarray following numerous manual washing and drying steps that have the potential to introduce methodological artifacts into the assay.

Using interaction data from the SPR assay as a low throughput gold standard approach [34], we tested 31 interactions and 32 non-interactions as predicted by FP. 24 of the 31 interactions replicated by FP while only 1 of the 32 non-interactions was determined to be an interaction by SPR (Table S7). From this, we estimated an FP false positive rate (FPR) of 18.4%, a false discovery rate (FDR) of 22.6%, and a false negative rate (FNR) of 4% (see Methods). Previously, PMs have been suggested to result in a quality of interaction data similar to the SPR methodology [34]. However, these comparisons were performed by examining a biased subset of only eight interactions by SPR that had previously been determined by PMs to be high affinity interactions. More recently, PMs have been purported to have a FPR and FNR of approximately 14%. However, these PM estimates were derived from cross-validating PM data using a single replicate of the FP assay and assuming it had 100% sensitivity to identify an interaction [43]. Our assessment of 19 true interactions identified by FP that validated by SPR indicated that queries in a single FP run resulted in the detection of only 63.3% of interactions. We speculate that the modest detection rate for a single pass of FP was likely the result of two factors: 1) batch-to-batch protein expression variability resulting in differences in SH2 domain protein activity; and 2) interaction affinities being sufficiently weak such that the variability in SH2 domain activity would result in an interaction call in one run but a non-call in a subsequent run. Consistent with this hypothesis, our intra-FP run detection reproducibility was high when only a single protein expression batch replicate was used. Given that we used identical protein expression protocols in our FP pipeline that were used in previous PM and FP studies [34], [35], [43], the batch-to-batch variability in protein activity is likely to have been similar across the studies. In short, using a single FP query would likely have resulted in incorrectly estimating the true FPR and FNR of PMs [43].

We also compared the FP-derived interactions between ErbB family members and the assayed proteins to a set of 44 cellular interactions found in the iRefWeb, BioGRID, HPRD, and IntAct resources (see Supplementary Methods, Table S8). While this comparison was not ideal given that these databases do not indicate the particular phosphosite responsible for the interaction, it provided a starting point for comparison with biologically relevant interactions. 40 of 44 (88.9%) known *in vivo* interactions were identified by our FP assay. Notably, our assay predicted more than 200 novel protein interactions (among greater than 1100 novel peptide-protein interactions).

### Competitive Displacement by Non-labeled Phosphopeptides Demonstrates Binding Pocket Specificity of FP-derived Interactions

**Figure 3:**
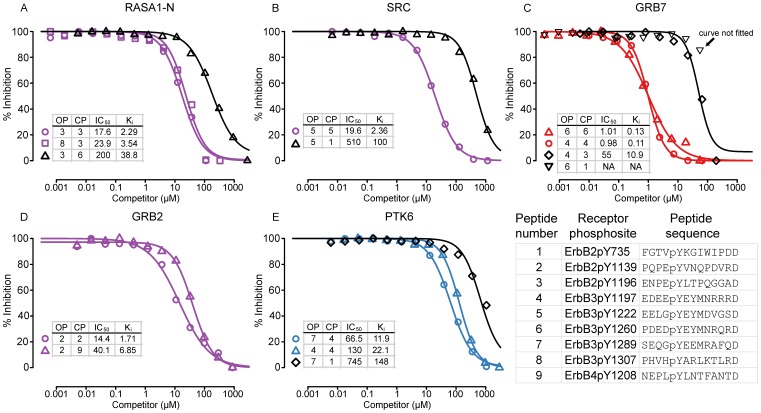
Competitive inhibition binding curves of protein-peptide interactions detected by FP. Nine ErbB phosphotyrosine sites were queried against five proteins: (**A**) RASA1-N, (**B**) SRC, (**C**) GRB7, (**D**) GRB2, and (**E**) PTK6. The predicted binding affinities of competitor peptide curves are color-coded as follows: red (*K_D_<*1), purple (1≤ *K_D_<*5), blue (5≤ *K_D_<*20), and black (*K_D_* ≥20). “OP” refers to the original rhodamine-labeled peptides and “CP” to the unlabeled competitor peptides, which have been numbered in the figure with sequences.

In order to verify that FP-derived interactions represented specific interactions between ErbB derived peptides and SH2 domain binding pockets, we tested a subset of interactions that included 13 SH2 domains and 10 unique ErbB-family phosphopeptides using a competitive displacement assay. In order to carry out this displacement analysis, we first identified a subset of interactions that would result in sufficient FP signal to allow for a statistically rigorous assessment of competitive displacement by non-labeled peptides. For this purpose, a representative set of 33 binary interaction partners was selected for Z’ factor analysis (see Supplementary Methods, Table S9) [44]. The Z’ factor is a statistical parameter that describes assay quality in terms of both signal strength and assay variation. The chosen interaction partners spanned a wide range of interaction affinities, were detected from at least three independent FP assay runs, and displayed an observed maximum polarization value (MaxP) greater than the bottom quartile of all interaction data (at least four-fold higher than the polarization cutoff threshold). 25 of the 33 (76%) peptide-protein interactions fell into the “excellent” class with Z’ factors ≥0.5 while the remaining eight (26%) fell into the “marginal” class [44] (Table S9). Eight protein-peptide interactions with Z’ factors >0.5 spanning a wide range of K_D_s (from ∼100 nM –6 µM) were then selected as a base set for subsequent competitive displacement analysis (Table S10). In total, 14 competition experiments were performed utilizing a matrix of five representative SH2 domains, seven labeled peptides, and six non-labeled competitor peptides. For all interactions tested, non-labeled phosphopeptides that were predicted to interact with an SH2 domain with K_D_<20 µM competitively displaced labeled peptides (Figure 3). Likewise, non-labeled versions of peptides not predicted to interact (K_D_ >20 µM) resulted in negligible competition. For example, ErbB3-pY1197 (Peptide 4) competitively displaced the labeled version of itself as well as ErbB3-pY1289 (Peptide 7) from the PTK6 SH2 domain (Figure 3E). Conversely, ErbB2-pY735 (Peptide 1), a peptide predicted by FP not to interact with PTK6 at a K_D_ less than 20 µM, did not displace half of ErbB3-pY1289 until titrated to a concentration of 148 µM (Figure 3E), thus validating this interaction as a true negative. The competitive binding assay provided additional evidence that interactions indicated by FP with a K_D_<20 µM were true binding-pocket mediated interactions and that interactions not detected by FP were true negatives. This result is important because it suggests the potential existence of many biologically relevant binary protein interactions that would be too weak and dynamic to be detected by standard methods such as co-immunoprecipitation.

### Comparison of High-throughput Fluorescence Polarization Assay and Protein Microarray Approaches

**Figure 4:**
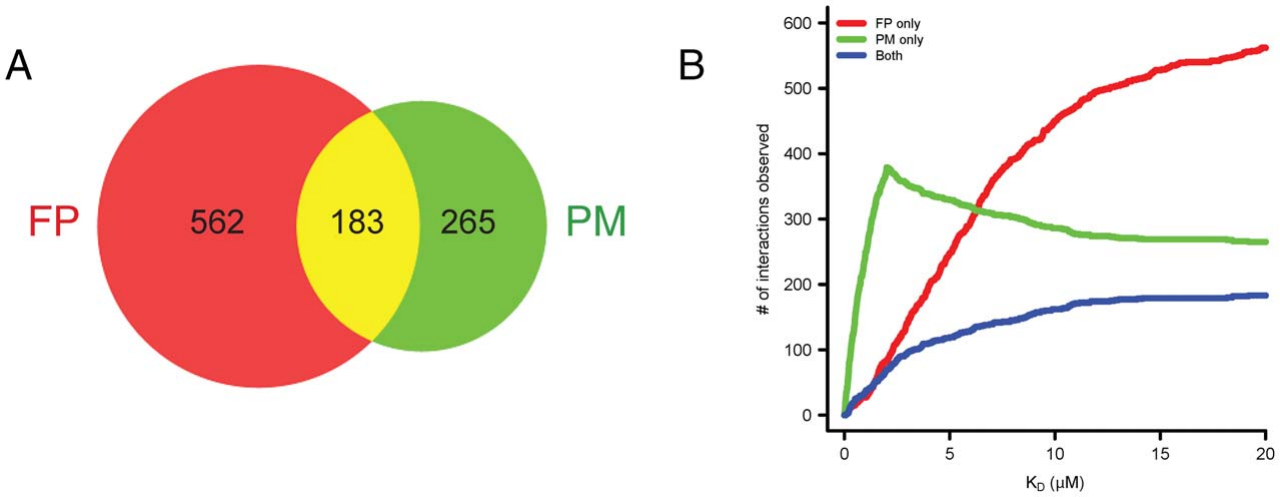
Methodological cross-comparison of SH2 interactions determined by fluorescence polarization or in published protein microarray data for the ErbB family. (**A**) Venn diagram comparison of overall SH2 recruitment profiles revealed by FP and PMs for the ErbB family of RTKs for only peptides and proteins tested by both platforms. The red circle represents protein-peptide interactions observed by FP; the green circle represents protein-peptide interactions previously observed by PMs; and the yellow overlap represents interactions observed by both methods. (**B**) SH2 and ErbB interactions quantified over a range of binding affinity thresholds as determined previously by PMs and in this study by FP data. The red line represents interactions characterized exclusively by FP; the green line represents interactions characterized exclusively by PMs; the blue line represents interactions observed by both methods.

We next compared the interaction overlap between previous PM datasets and the FP interaction matrix. For these analyses, we limited our comparison to the most recent estimate of 448 protein interactions representing 462 unique ErbB phosphotyrosine site interactions stemming from three previously published PM studies (Table S6, Figure S5)[34]–[36]. We included only interactions derived from the 88 unique proteins and 54 unique phosphopeptides that were examined by both PMs and FP. 183 of the 448 interactions previously identified by PMs overlapped with the 745 interactions determined by FP (Figure 4A, Figure S6). Analysis of the FP derived data indicated fewer high affinity interactions (K_D_<2 µM) than suggested by previous PM studies and a much greater number of moderate-to-low affinity interactions (20> K_D_ >2 µM) (Figure 4B). We also observed a trend for tighter PM interactions to be more likely to be replicated by our FP assay (Figure S7). The tightest quantile (K_D_≤0.3 µM) of PM interactions was over 1.5 times more likely to be identified by FP as compared to the weakest quantile (K_D_≥1.28 µM) (59/112 vs. 38/112, χ^2^ test *P* = 0.007).

Given that our FP dataset has a FNR of 4% based on SPR cross-validation, we sought to estimate the true false discovery rate (FDR) of PMs by identifying the proportion of PM interactions that were not detected by FP. 265/448 (59%) interactions detected by PMs were not called as interactions in our FP data set. To estimate the true FNR of PMs, we next established a set of 235 “high-confidence” FP interactions whose observed replication rates significantly exceeded the expected rates given their K_D_ (*P*>0.5, see Supplementary Methods and Table S11). This subset of interactions excludes many real interactions but has a low FPR (calculated to be 3% from comparison to SPR cross-validation results, Figure S8). We estimated the true PM FNR by examining the proportion of high-confidence FP interactions that were not detected by PMs (149/235); assuming that interactions identified independently by both FP and PM are true positives, this suggests a PM FNR of 45% (149/332). Together, these results provided evidence that previous reports may have substantially underestimated the FPR and FNR values of PMs [43].

In order to more systematically compare the technical reproducibility of the FP and PM methods, we performed 39 in-house PMs using 12 phosphopeptides. We then compared the reproducibility of identified interactions from PMs with the reproducibility from the FP assay (Table S12). A similar proportion of “intra-assay” interactions overlapped between PMs and FP assays: peptides probed against domains from the same batch of printed microarrays generated similar reproducibility as peptides probed against SH2 domains within the same FP run (70.6% (346/490) for PMs vs. 71.8% (275/383) for FP; χ^2^ test *P* = 0.76). However, when interaction data was assessed from PMs fabricated at different times, the reproducibility of PMs was much less compared to data taken from FP performed on different days (34.2% vs. 55.9%; 226/660 vs. 2812/5028, χ^2^ test *P*<2.2×10^−16^) thus indicating substantial batch to batch variability in the PM methodology and that technical replicates are critical to obtain an estimate of the veracity of interactions and non-interactions called by PMs.

### Elucidation of Common and Distinct Recruitment Locations of SH2 Domains within ErbB Receptors

Analysis of the interaction dataset revealed trends in the recruitment locations of different SH2 domains with ErbB receptors. Although some SH2 domains like those from PLCG1 were recruited to many phosphosites in each receptor, others were recruited to only a small number of phosphosites located in the same general location in each ErbB family member (Figure 2, Table S4). For example, SHD1A and SH2D1B were recruited with high affinity (in the nM KD range) to one membrane proximal phosphosite in each receptor (ErbB1pY727, ErbB2pY772, ErbB3pY789, and ErbB4pY733). Tensin family SH2 domains (e.g. TENC1, TNS1, and TNS3) were recruited with relatively high affinity to two membrane proximal phosphosites (pY764 and pY801) in ErbB1 and one membrane proximal phosphosite (pY823) in ErbB3. Similarly, each receptor recruited N-terminal PIK3R family SH2s with high affinity (in the nM K_D_ range) to one central phosphosite in addition to differentially recruiting these domains with lower affinity to receptor sites at other locations. VAV GEF family SH2 domains were recruited with highest affinity to one central and one C-terminal site of ErbB3 (in nM K_D_ range), and to centrally located phosphosites of ErbB1, ErbB2, and ErbB4. SH2 domains from SRC and other tyrosine kinase genes were recruited with moderate affinity (in 1 µM K_D_ range) to both N- and C-terminal sites of ErbB3 and to similar locations but at lower affinity to ErbB4; these domains were recruited to C-terminal phosphosites of ErbB1 and ErbB2 with much lower affinity (in 10–20 µM K_D_ range). GRB2 and GRAP2 SH2 domains were recruited with moderate affinity (in the 1 µM K_D_ range) to a single C-terminal phosphosite of ErbB4, to one membrane proximal and one C-terminal phosphosite within ErbB2, and to two C-terminal phosphosites within ErbB1. We speculate that the locations of domain recruitment to ErbB receptors may be important in the assembly of receptor specific signaling modules.

### ErbB Family Recruitment Capacity at Different Affinity Thresholds

**Figure 5:**
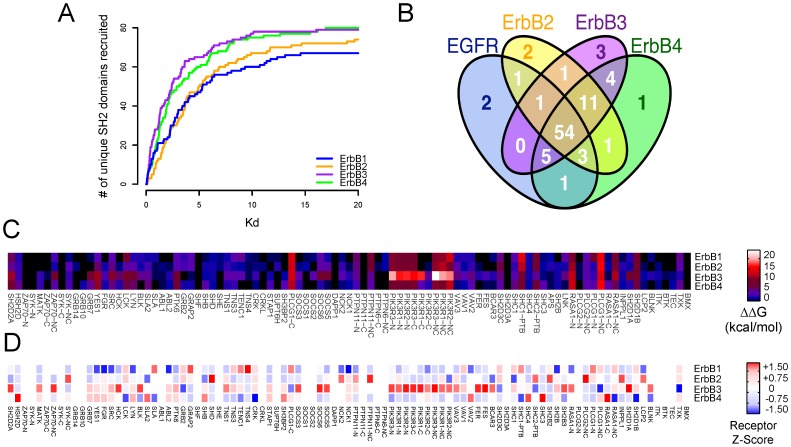
Characterization of unique and overlapping SH2 domain recruitment patterns by individual ErbB receptors. (**A**) SH2 recruitment potential of ErbB1 family members at different affinity thresholds. The total number of unique SH2 and PTB domains recruited over a range of affinity thresholds are depicted for each receptor. (**B**) Four-way Venn diagram (not to scale) depicts SH2 domain interactions shared by or exclusive to ErbB1, ErbB2, ErbB3, and ErbB4. (**C**) Relative binding free energies of interactions described in Figure 2 are summed for each ErbB receptor. (**D**) Relative enrichment and depletion of binding sites for recruitment of each SH2 domain by each ErbB receptor, depicted by Z-score transforming the observed number of binding sites each receptor had for a particular domain relative to the average number of sites that bound that domain across all ErbB receptors. Domains recruited by fewer than four independent pY sites were excluded from this analysis.

Previous PM-based studies have suggested that the oncogenic potential of ErbB2 arises because of its unique ability to promiscuously recruit many different SH2 domains with low affinity [34]. The over-expression of ErbB2 in the context of cancer might then bring about the aberrant recruitment of many SH2-containing proteins, thus eliciting increased mitogenesis, migration, and survival. When we examined the interaction promiscuity of each ErbB family member in light of the new FP-derived data, we found that ErbB2 did not recruit a higher number of SH2 domains than other receptors as the affinity threshold was relaxed to include weaker interactions (Figure 5A). By contrast, ErbB3 and ErbB4 recruited more SH2 domain-containing proteins than ErbB1 and ErbB2 across all interaction strengths; moreover, this trend was maintained when we filtered for only the highest-confidence interactions in our data set (Figure S9). At the lowest affinity threshold, ErbB1, ErbB2, ErbB3, and ErbB4 recruited 66, 74, 79, and 80 unique domains respectively, 54 of which were commonly recruited to all receptors (Figure 5B). Several factors are likely to account for this discrepancy between PMs and the FP method: 1) the PM experiments queried only a small subset of all ErbB phosphotyrosine sites; 2) the higher FPR of PMs owing to irreversible disulfide bonding of peptides to protein domains in the absence of reducing agent; and 3) the inability of the PM method to reproducibly query interactions with affinities weaker than 2 µM K_D_s because of aggregation of a subset of rhodamine labeled peptides when titrated at concentrations exceeding 1 µM. Notably, although ErbB4 has more total intracellular tyrosines compared to the other receptors (29 for ErbB4 versus 20, 19, and 24, for ErbB1, ErbB2, and ErbB3, respectively), it had the most peptides that showed no interactions with any SH2 domains, perhaps owing to its additional transcription related functions in the nucleus [45], [46]. Seven queried ErbB4 peptides resulted in no interactions versus one peptide for ErbB1, one peptide for ErbB2, and none for ErbB3.

### Comparison of SH2 Recruitment Capacity Among ErbB Receptors

In order to further define and classify commonalities and differences among the ErbB family, the relative binding free energy (FE) of each SH2 domain was summed across all phosphosites on each receptor relative to the assay detection threshold of 20 µM according to Equation (3):
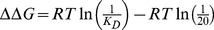
(3)


After summation, the common and distinct recruitment capacity of each ErbB receptor for SH2 domains was more readily apparent (Figure 5C, Table S13). ErbB1 and ErbB2 each recruited the SHC1 PTB domain with about 1.5 fold the relative binding FE of either ErbB3 or ErbB4. ErbB3 displayed higher binding FE for SH2 domain proteins from PIK3R, RASA1, and BCAR3 genes, as well as SH2 domains from VAV, SRC, and SOCS gene families versus the other receptors (Figure 5C). ErbB3 and ErbB4 each recruited PTK6 with more than twice the binding free energy of either ErbB1 or ErbB2. Additionally ErbB3 and ErbB4 each recruited the adapter proteins GRB7, CRK, BRDG1, SHB, and SH3B2 with two- to ten-fold higher binding FE than either ErbB1 or ErbB2. In contrast to PMs which suggested that GRB7 interacts at high affinity with many phosphopeptides from all receptors [34]–[35] (Figure S5), the FP data suggested high affinity binding sites (K_D_s ≤100 nM for Y1197 and Y1260) existed exclusively on ErbB3, consistent with a previous report demonstrating the ability of peptides derived from specifically ErbB3-pY1197 and -pY1260 to capture GRB7 from cell lysates [47]. While ErbB3-pY1260 was queried by PMs, no interaction was detected with GRB7. Conversely, the peptides corresponding to ErbB2-pY1221 and pY1222 were queried on both FP and PM platforms but only displayed interactions with GRB7 from the PM platform (Figure 2, Figure S5).

Strikingly, although PIKR3-C terminal SH2 domains bound with substantial FE to all ErbB receptors, the PIK3R1 and PIK3R2 C-terminal SH2 domains were selective and bound ErbB3 with 4- to 50-fold higher FE than ErbB1, ErbB2, and ErbB4. In the context of a cell, the tandem SH2 domains of PIK3R3 would be expected to bind with relatively high affinity to all ErbB receptors through avidity gained by binding with both domains. Conversely, PIK3R1 and PIK3R2 would display avidity for only ErbB3 and not the other receptors because their C-terminal domains bind only ErbB3 sites with high affinity. The highest affinity PIK3R recruitment sites on ErbB1, ErbB2, and ErbB4 that were determined by FP (which contained the canonical pYMXM PI3KR consensus binding sequence) were not previously examined by PMs. The C-terminal PIK3R1 and PIK3R2 SH2 domains rarely interacted with any phosphopeptides when probed with PMs indicating that the domains may have potentially lost functionality when immobilized on the microarray surface.

In addition to displaying differences in total affinity of recruitment of domains, we found that some receptors were enriched for total numbers of binding sites versus other receptors. We found that ErbB1 was enriched for total SHC1 PTB, and PLCG1 and TNS family SH2 domains versus the other receptors but depleted versus the other receptors for binding sites for SRC and NCK family SH2 domains (Figure 5D; Table S14. ErbB2 was enriched for INPPL1 inositol phosphatase, the PTPN11 phosphatase, and NCK family SH2 domain binding sites. ErbB3 was enriched for SH2D2A and BCAR3 binding sites as well as SRC, SOCS, and PIK3R family binding sites (Figure 5D, Table S14). However, ErbB3 was depleted for binding sites for domains from PLCG1 and PLCG2 genes which may further potentiate phosphatidyl inositol kinase signaling due to lack of recruitment of phospholipase activities. ErbB4 was depleted compared to other receptors for SH2D1, TNS, CRK, GRB2, and PI3KR family SH2 domain binding sites.

### Molecular Functions Recruited by ErbB Receptor Family Members

**Figure 6:**
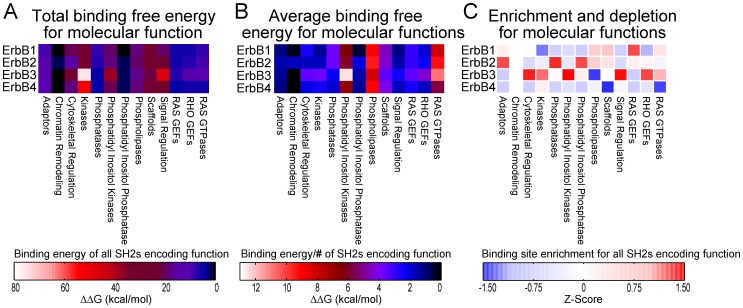
Comparison of the affinity with which each ErbB family member recruits proteins representing several molecular function categories. Relative binding free energies of interactions described in Figure 5C were summed across all domains contained in a particular functional category [55] in (**A**) (see Table S16) and divided by the number of SH2 domains represented in each class in (**B**) to determine an average recruitment potential for SH2s from each functional class. (**C**) ErbB receptor enrichment and depletion for binding sites for functional groups depicted by Z-score transformation of the raw data as in Figure 5D.

To better understand SH2 domain recruitment by ErbB receptors in the context of receptor biology, we clustered the domains into groups based on several key molecular function categories such as cell proliferation, migration, and survival (Figure 6) [48] (http://www.geneontology.org/; Table S15). We then summed the FE for each functional class (Figure 6A) and normalized the data by the number of SH2 domains in the particular functional class in order to assess each receptor’s average recruitment strength for each functional class (Figure 6B; Table S16). Overall, ErbB receptors recruited domains from phosphatidyl inositol kinases, phospholipases, and RAS GTPases with a higher FE per protein than from other functional groups. When compared to one another, ErbB3 displayed significantly higher binding free energy for domains from the RHO-GEF function than did ErbB1 or ErbB2, and displayed significantly higher binding free energy for domains from tyrosine kinases, phosphatidyl inositol kinases, and signal regulation functions than the other receptors (Figure 6A, Figure S10, Table S16); however, ErbB3 displayed significantly lower FE for scaffolds. ErbB1 had significantly lower FE for domains from the adapter category versus the other receptors whereas ErbB2 displayed significantly lower FE for domains from proteins involved in cytoskeletal regulation. ErbB1 and ErbB2 displayed nearly twice the binding FE for domains from the phosphatase ontology versus ErbB3 and ErbB4.

We also examined whether a significant enrichment existed for the total number of binding sites contained by each receptor for domains from particular molecular function categories (Figure 6C, Table S14-Tab2). ErbB2 was enriched for total phosphatase (*P* = 0.01) and phospholipase (*P* = 0.03) binding sites. ErbB3 was enriched for cytoskeletal regulation (*P* = 0.049), kinase (*P* = 0.001), phosphatidyl inositol kinase (*P*<1×10^−5^), and signal regulation (*P* = 0.002) binding sites. ErbB3 also recruited significantly more unique tyrosine kinases than the other receptors (*P = *0.04): 20 for ErbB3 versus 11, 16, and 17 by ErbB1, ErbB2, and ErbB4, respectively.

## Discussion

In the first phase of this study, we rigorously assessed several technical parameters of the FP method including analysis of false positive and false negative rates through cross-methodological comparison with the SPR assay. We additionally validated the binding pocket specificity of interactions identified via the FP method by performing competition binding analyses with unlabeled phosphopeptides. Our study captured 1405 unique biological interactions, 1169 (83.2%) of which were not described by previous PM studies. This increase in interactions captured by the FP method was likely attributable to: 1) querying a 50% larger peptide set than previous studies; and 2) the high rate of true positives and low rate of false negatives of the FP method relative to PMs or other commonly used high-throughput protein-protein interaction analysis methods. Our analysis of PMs indicated a higher rate of false positives and false negatives than has been previously reported. Of 656 SH2-phosphopeptide interactions reported to date by PMs, only 236 were confirmed by the FP assay.

We speculate that the following reasons may contribute to the higher rate of PM false negatives. Firstly, the microarray printing of PM slides typically requires a period of about ten hours followed by an additional several hours of dehydration prior to freezing. These steps may potentially lead to reduced functionality of printed proteins. Secondly, immobilization of SH2 domains via amine chemistry on aldehyde-coated slides may further reduce the functionality of printed proteins. Lastly, PM platforms cannot accurately measure peptide interactions with K_D_s weaker than about 2 µM because of the tendency of rhodamine-labeled peptides to aggregate resulting in visual artifacts on the array surface [34]. By contrast, the FP method is performed by measuring the interaction of rhodamine-labeled peptides at a constant 10 nM in the solution phase while being queried for interactions with SH2 domain proteins titrated from low to high concentrations. We have encountered no technical difficulties related to protein precipitation following titration of SH2 domains up to concentrations of 100 µM. Thus, it is likely that many high-confidence, but low-affinity interactions that were determined by FP could not be identified by PMs because of peptide precipitation.

With regard to PM false positives, we suspect that non-specific interactions may arise from denaturation of proteins during surface immobilization, incomplete solubilization of either aggregated or precipitated peptides, and from non-specific interactions between peptides and microarray slide blocking agents such as bovine serum albumin or gelatin. The PM method requires that microarrays be washed with sufficient rigor to remove non-specifically bound peptides from proteins and glass without dissociating peptides specifically bound to SH2 protein spots. Peptides that bind more weakly to particular proteins are more likely to be lost during the wash procedure than high affinity binders because of higher off rates and lower total occupancy of protein spots at a given concentration. At sufficiently high concentrations, most peptides will interact non-specifically with blocking agents. Noise generated from such interactions places practical limits on the strength of interactions that can be queried with PMs.

False positive interactions may also result from disulfide bonding of SH2 domains with one another and/or receptor peptides leading to a reduction in the kinetics by which non-specifically bound peptides unbind and wash away into bulk solution. Previous SH2 PM experiments have been performed in the absence of reducing agents, whereas FP experiments in this study were performed with dithiothreitol (DTT). Since the majority of intra-cellular compartments are reducing in nature, we hypothesized that the inclusion of a reducing agent would represent a more physiologically relevant environment for assaying SH2-ErbB interactions. When we included DTT with peptides on PMs, we observed no loss in Cy5 signal originating from immobilized protein domains. However, we saw a dramatic reduction in rhodamine signal from bound peptides washed under identical conditions (Figure S11). Although DTT had the greatest effect on cysteine-containing ErbB peptides probed with PMs, it also resulted in a substantial reduction in rhodamine-derived signal from non-cysteine containing peptides (Figures S12 and S13; Table S17) assayed by PMs. Many SH2 domains have one or more cysteines that could mediate inter-protein disulfide bonding. The resulting reduction in rates of diffusion of peptides into bulk medium during the wash procedure may explain the increases in peptide signal following PM washing in the absence of DTT. The previous PM study [34] was cross-validated via SPR analysis with a sample size of only eight interactions. These interactions were selected non-randomly and were all of relatively high affinity (<2 µM). For our current FP study, we performed cross-validation in a more rigorous way by randomly selecting a larger number of interactions which spanned a much wider range of interaction affinities (0.26–15.49 µM).

In the second phase of this study, we explicitly compared the reproducibility of data obtained by the FP method with that obtained by PMs. Publicly available PM datasets have aggregated new interaction data with old data without indicating which was new or old [35], [36]. Statistical analysis of reproducibility metrics using such data was therefore impossible. We therefore performed a series of controlled in-house PM experiments specifically aimed at assessing reproducibility. Within batches of slides that were fabricated simultaneously, the reproducibility of data obtained by PMs was similar to the FP method. However, when comparing PMs between batches fabricated at different times, the reproducibility was much lower than the FP method. Unlike PMs, which required multiple steps of human intervention following protein expression and purification including microarraying, desiccation, freezing, thawing, blocking, washing, image analysis, and spot finding, no human intervention was required for the FP method following the physical placement of plates containing peptides and proteins onto the automated pipetting and analysis deck. This lack of human intervention in the FP workflow coupled with the solution phase nature of the assay likely contributed to the greater reproducibility of interactions determined across different FP runs versus the reproducibility of interactions characterized across batches of printed microarrays.

We estimate that the greatest source of variability in the FP assay, which similarly contributes to variability in the PM assay, is batch-specific differences in protein functionality. In our FP study, the amount of protein obtained for some domains from a single 500 ml bacterial expression culture was often not sufficient for querying all peptides in our dataset multiple times. This problem could be mitigated to a certain extent by making larger batches of recombinant protein. However, difficulty in estimating the absolute concentrations and relative functionality of all of the protein domains from a single expression would still render the resulting apparent K_D_s semi-quantitative estimates of the true values. For these reasons, we favor an approach whereby each protein is expressed in multiple batches and independently tested for interaction with phosphopeptides multiple times. The average affinities can then be examined with a better perspective regarding protein functionality and assay variability.

In the third phase of this study, we used the data obtained from the FP method to assess the interaction of phosphopeptides corresponding to nearly every intracellular tyrosine of all four human ErbB receptors with most of the genomically encoded human SH2 domains. Although previous studies have indicated that tyrosines reported to be phosphorylated in the literature have a higher probability of generating interactions with SH2 domains [35], [36], our data suggested no such trend. Of the 89 peptides in our study, 57 were previously reported to be phosphorylated in the literature and 32 were not; of these, we directly tested 53 and 21 peptides, respectively for interaction by FP. 390 out of 1995 (19.5%) possible interactions were identified from tyrosine-containing peptides not previously reported to be phosphorylated whereas 883 out of 5035 (17.5%) possible interactions were identified for the group of peptides that was previously reported to be phosphorylated in the literature (Table S3) [18], [20], [21]. Rather, we observed a statistically suggestive trend in the opposite direction, indicating that sites not previously reported to be phosphorylated were responsible for a slightly higher proportion of interactions (χ^2^ test *P* = 0.052). Indeed, membrane-proximal phosphosites such as ErbB1-pY813, ErbB2-pY685, ErbB3-pY789, ErbB3-pY823, and ErbB4-pY906 reproducibly interacted at high affinity with many SH2 domains, but have not previously been reported to be phosphorylated (Table S3). For example, ErbB1-pY813 interacted with tensin family SH2 domains; ErbB2-pY685 interacted with SOCS family SH2 domains; ErbB3-pY823 interacted with Src kinase and tensin family SH2 domains; and ErbB4-pY906 interacted with ZAP70, GRB7, and GRB10 SH2 domains (Table S11, Figure S14).

Although most ErbB receptor auto-phosphorylation sites have been reported in the literature numerous times (with several being reported over 25 times), many hetero-phosphorylation sites–those that require the kinase activity of a non-receptor tyrosine kinase–have seldom been reported in the literature (less than five times) (Table S3). The fact that many of these phosphorylation events have been reported in a single mass spectrometry literature source is evidence of their existence. Our observation that these sites are just as likely to result in an SH2 domain interaction as sites not reported to be phosphorylated further implicates their importance in biologically relevant functions. A standard method used in molecular biology field for characterization of phosphorylation events on RTKs consists of examination of tyrosine phosphorylation events in a cell with high RTK expression following growth factor stimulation of a cell line [49]–[51]. We hypothesize that alternative targeted approaches that are more sensitive at distinguishing the phosphorylation of ErbB receptors in subcellular compartments at physiological ErbB receptor levels would allow for a greater probability of characterizing heterophosphosites that are likely to occur at low stoichiometry and in a manner that is dependent upon colocalization with particular non-RTKs. Ideally, affinity reagents could be generated against potential heterophosphosites so that they can be examined with high-throughput immunoblotting approaches under a variety of conditions [52] or with immunostaining techniques such that spatial distribution of phosphorylation can be appreciated on a cell to cell basis.

About 80% of the interactions determined by FP displayed a K_D_ weaker than 2 µM (Figure S15). While it is tempting to dismiss these interactions as noise or non-physiological, all predicted positive interactions in our test set, regardless of affinity, were validated to represent specific binding pocket-mediated interactions that were able to be competitively displaced by other non-labeled peptides, even when the competitor peptides also were suggested by the FP assay to bind with similarly low affinities (Figure 3). Indeed, many of these low affinity interactions were high-confidence interactions in that they were found to replicate more than expected across multiple, independent FP runs performed with protein batches that were independently expressed and purified. Additionally, several interactions between SH2 domains and phosphosites with known biological relevance have previously been shown to have similarly weak affinities [37]. The highest affinity interaction between the c-Src SH2 domain and ErbB1 in our FP assay was in the 4 µM K_D_ affinity range and provided further evidence in support of the hypothesis that many biologically relevant interactions between ErbB receptors and SH2 domains may be of relatively low affinity and too weak to be detected in cells by standard cell biological methods such as immunoprecipitation.

By displaying the interaction data for each receptor phosphosite as a percentage of the total receptor recruitment, we constructed a map that should be useful for future site-directed mutagenesis experiments aimed at demonstrating the relevance of these interactions in cells (Figure S16). For example, our data suggested that ErbB1 tyrosines 1092 and 1138 are each responsible for 50% of the binding FE for GRB2 and GRAP2. Similarly tyrosines 801, 813, and 900 each contributed 20% to 60% of the total binding free energy for tensin family SH2 domains. Informed combinatorial mutagenesis of key ErbB phosphosites should thus reveal the role of specific domains in a biological context.

Systems-level assessment of the network map revealed several insights that were not previously appreciated. Firstly, our analysis suggested that all receptors displayed similar trends in interaction promiscuity as the affinity threshold was lowered to include weaker interactions. However, they also displayed quantitative differences in the number of recruitment sites for particular SH2 domains, the affinity of recruitment of SH2 domains, and the general locations to which SH2 domains were recruited. This analysis contrasted with previous PM based findings which had suggested that ErbB2 recruited many more proteins at low affinity than ErbB1, ErbB3, or ErbB4 [34]. Additionally, in contrast to previous findings based on PMs [35], we found no evidence that ErbB4 phosphosites are unusually selective in their domain interaction versus other receptors.

Heterodimers of ErbB2 and ErbB3 have been suggested to contain the highest growth and transforming potential of any dimer combination [53], [54]. We hypothesize that the increased SH2 domain recruitment capacity of such heterodimers might also be responsible for increased transformation potential. Consistent with this hypothesis, heterodimers of ErbB2 and ErbB3 were found to recruit the largest set of agonistic signaling molecules. While ErbB2 recruited phospholipase and MAPK activators (GRB2, GRAP2, and SHC proteins) with the highest binding free energy of any of the receptors, ErbB3 recruited SH2 domains from the phosphatidyl inositol kinase, RHO GEF, signal regulation, and cytoskeletal regulation ontologies with higher binding free energy than the other receptors and recruited phosphatases with lower binding free energy than either ErbB1 or ErbB2. In addition, ErbB3 and ErbB4 were the only receptors with high affinity membrane-proximal recruitment sites for tyrosine kinases. Such recruitment may contribute to the enhanced phosphorylation of other membrane proximal heterophosphosites and for the subsequent membrane-proximal recruitment of other signaling proteins such as the tensin family, CRK, and SH2D1A proteins.

The biochemical interactions uncovered in this assay are the result of a large scale *in vitro* screen to comprehensively query the interaction potential of every intracellular tyrosine of every ErbB receptor. The true biological meaning of these interactions is still unknown. However, a wealth of novel interactions is contained in this dataset which represents the SH2 domain biochemical recruitment potential of the ErbB receptors. This data should serve as informed testable hypotheses for targeted follow up experiments aimed at further characterization of ErbB receptor mechanisms in health and disease.

## Supporting Information

Figure S1 **Analysis of SH2 and PTB domain purity following expression and purification.** Recombinant proteins were spectrophotometrically normalized to a concentration of ∼20 µM, electrophoresed by SDS-PAGE and stained with GelCode Blue (Pierce). A representative set of all assayed proteins used in this study is displayed.
(PDF)

Figure S2 **Short-form peptides produce higher polarization values than long-form peptides.** (**A**) Heatmaps depict apparent midpoint dissociation constants (K_D_s) of SH2 and PTB domains with indicated 18-mer (upper panel) and 13-mer (lower panel) peptides. KDs are color coded by affinity (see scale). Lower-case “d” denotes aspartic acid (Asp) residue pre-charged on the peptide synthesis resin and not a naturally-occurring Asp. (**B**) Polarization values obtained by FP were fit to equation (1) and the theoretical maximum polarization values induced by SH2 and PTB domains were displayed as heat maps. (**C**) Side-by-side 13-mer and 18-mer bean plots of Pmax values.
(PDF)

Figure S3 **Histogram depicting how many times each peptide-protein interaction was tested across all six automated FP runs in our assay.**
(PDF)

Figure S4 **Heatmaps depicting maximum polarization values of interactions of SH2 and PTB domains with all ErbB family phosphopeptides.** Polarization values obtained by FP were fit to equation (1) and the theoretical maximum polarization values induced by SH2 and PTB domains were output as heat maps. Identical peptides among the four receptors are indicated numerically. Lower-case “d” refers to the aspartic acid (Asp) residue pre-charged on the peptide synthesis resin and not a naturally-occuring Asp. Peptides that were queried but resulted in no positive interactions are designated “NI”; peptides that were unable to be synthesized are designated “NS”. Rows in the heatmaps for these peptides are greyed out to indicate that the assay could neither confirm nor deny positive or negative interactions from these peptides.
(PDF)

Figure S5 **SH2 domain recruitment potential of the ErbB family as previously determined by protein microarrays.** Color-coded heat maps (see legend) represent apparent dissociation constants (K_D_s) for protein microarray interactions between SH2/PTB domains and phosphopeptides representing potential ErbB1, ErbB2, ErbB3 and ErbB4 phosphotyrosine sites. Binding strength is color-coded as indicated on the legend. Homologous peptides with identical amino acid sequences at the +1 to the +4 position relative to the phosphotyrosine (X) are marked with an asterisk followed by the number of the homologous receptor with sequences indicated. Lower-case “d” denotes the aspartic acid (Asp) residue pre-charged on the peptide synthesis resin and not a naturally-occurring Asp. Rows of the heatmaps for peptides that have no previously reported protein microarray interactions are grayed out to indicate that no experiments were performed to confirm or deny positive or negative interactions from these peptides.
(PDF)

Figure S6 **Overlap between protein microarrays and fluorescence polarization approaches.** Interactions detected by protein microarrays (PMs) in previous studies are plotted with interactions detected by the fluorescence polarization (FP) assay in this study. Interactions detected by PMs alone are colored green. Interactions detected by FP alone are colored red. Interactions detected by both platforms are colored in yellow. Homologous peptides with identical amino acid sequences at the +1 to the +4 position relative to the phosphotyrosine (X) are marked with an asterisk followed by the number of the homologous receptor with sequences indicated. Lower-case “d” denotes the aspartic acid (Asp) residue pre-charged on the peptide synthesis resin and not a naturally-occurring Asp. Peptides that were unable to be synthesized (NS) and those that were queried but resulted in no positive interactions (NI) are indicated. Rows of the heatmaps for these peptides are grayed out to indicate that neither FP nor PM could experimentally confirm or deny positive or negative interactions from these peptides.
(PDF)

Figure S7 **Fluorescence polarization replication probability as a function of the strength of interaction as estimated by protein microarrays.** 448 interactions previously detected by protein microarrays were binned into 8 groups based on their interaction strengths and the proportion of interactions that were detected at least once by FP calculated.
(PDF)

Figure S8 **False positive rate (FPR) and false negative rate (FNR) as a function of the probability of each interaction calculated by comparing the observed versus the expected replication rate for an interaction given its strength.** As expected, filtering for “high-confidence” interactions (P>0.5) gives a low FPR at the expense of greatly inflating the FNR.
(PDF)

Figure S9 **Characterization of unique and overlapping SH2 domain recruitment patterns by individual ErbB receptors for high-confidence interactions only.** **(A)** Four-way Venn diagram depicts SH2 domain interactions shared by or exclusive to ErbB1, 2, 3, and 4. **(B)** SH2 recruitment potential of EGFR family members at different affinity thresholds. The total number of unique SH2 and PTB domains recruited over a range of affinity thresholds are depicted for each receptor.
(PDF)

Figure S10 **95% confidence intervals for total free binding energy for each receptor for each molecular function.** Relative binding free energies of interactions were summed across all domains contained from a particular function. Confidence intervals were calculated by summing ΔΔG +/−1.96 standard deviations derived from the distribution of K_D_ estimates of individual interaction replicates.
(PDF)

Figure S11 **Inclusion of DTT causes dramatic reduction of intensities of spots on protein microarrays following probing with both cysteine and non-cysteine containing phosphopeptides.** Shown are blocks of printed SH2 and PTB domains probed with 1 µM concentrations of indicated fluorescent ErbB peptide solutions (sequences indicated with phosphotyrosine designated by a red “X” and the asterisk denoting the N-terminal rhodamine label) containing 0 mM (-DTT, left panels) or 5 mM DTT (+DTT, right panels). Red spots (upper panels) indicate printed SH2 domains which were printed with trace amounts of Cy5-labeled bovine serum albumin for spot finding purposes during microarray analysis. Rhodamine-labeled peptides bound to arrayed SH2 domains are green in color (lower panels). Spot identities for several SH2 domains are indicated for interactions observed probing with the peptide derived from ErbB2-pY1139 (upper left quadrant of images).
(PDF)

Figure S12 **SH2 domain recruitment potential of the ErbB family as determined by protein microarrays in the absence of DTT.** Color-coded heat maps (see legend) represent apparent dissociation constants (K_D_s) for protein microarray interactions between SH2/PTB domains and phosphopeptides representing all potential ErbB1, ErbB2, ErbB3, and ErbB4 phosphotyrosine sites. Binding strength is color coded as indicated. Homologous ErbB peptides with identical amino acid residues from +1 to the +4 position relative to the phosphotyrosine (X) are indicated with an asterisk followed by the number (in order of occurrence) of the homologous receptor. Sequences of peptides used are indicated for each homologous receptor site. Lower-case “d” denotes the aspartic acid (Asp) residue pre-charged on the peptide-synthesis resin and not a naturally-occurring Asp. Peptides that resulted in no positive interactions are designated “NI”; peptides not synthesized for PMs are designated “NS”. Rows of the heatmaps for these peptides are grayed out to indicate that our FP assay could neither confirm nor deny positive or negative interactions from these peptides.
(PDF)

Figure S13 **SH2 domain recruitment potential of the ErbB family as determined by protein microarrays in the presence of DTT.** Color-coded heat maps (see legend) represent apparent dissociation constants (K_D_s) for protein microarray interactions between SH2/PTB domains and phosphopeptides representing all potential ErbB1, ErbB2, ErbB3, and ErbB4 phosphotyrosine sites. Binding strength is color coded as indicated. Homologous ErbB peptides with identical amino acid residues from +1 to the +4 position relative to the phosphotyrosine (X) are indicated with an asterisk followed by the number (in order of occurrence) of the homologous receptor. Sequences of peptides used are indicated for each homologous receptor site. Lower-case “d” denotes the aspartic acid (Asp) residue pre-charged on the peptide synthesis resin and not a naturally-occurring Asp. Peptides that resulted in no positive interactions are designated “NI”; peptides not synthesized for PMs are designated “NS”. Rows of the heatmaps for these peptides are grayed out to indicate that our FP assay could neither confirm nor deny positive or negative interactions from these peptides.
(PDF)

Figure S14 **High confidence SH2 domain recruitment potential of the ErbB family as determined by high-throughput fluorescence polarization (HT-FP).** Color-coded heat maps represent apparent dissociation constants (K_D_s) for FP interactions between SH2/PTB domains and phosphopeptides representing all potential ErbB1, ErbB2, ErbB3, and ErbB4 phosphotyrosine sites. Homologous ErbB peptides with identical amino acid residues from +1 to the +4 position relative to the phosphotyrosine (X) are indicated with an asterisk followed by the number (in order of occurrence) of the homologous receptor site. Sequences of peptides used are indicated for each homologous receptor site. Lower-case “d” denotes the aspartic acid (Asp) residue that was pre-charged on the peptide synthesis resin and not a naturally-occurring Asp. Peptides that resulted in no positive interactions are designated “NI”; peptides that were unable to be synthesized are designated “NS”. Rows of the heatmaps for these peptides are grayed out to indicate that our FP assay could neither confirm nor deny positive or negative interactions from these peptides.
(PDF)

Figure S15 **FP technical reproducibility and interaction number as a function of the strength of the interaction.** **(A)** The probability of an interaction detected by FP being identified in subsequent runs. **(B)** Histogram depicts the number of interactions identified at each affinity threshold in our FP assay.
(PDF)

Figure S16 **FP determined SH2 domain recruitment activity by ErbB receptors as a product of free energy potential.** Color-coded heat maps (see legend) depict free energy contributions for FP interactions between SH2/PTB domains and phosphopeptides representing all potential ErbB1, ErbB2, ErbB3, and ErbB4 phosphotyrosine sites. Binding strength is color coded as indicated and represents free energy as a percentage of total recruitment potential of an SH2/PTB across all ErbB peptides for a particular receptor. Homologous ErbB peptides with identical amino acid residues from +1 to the +4 position relative to the phosphotyrosine (X) are indicated with an asterisk followed by the number (in order of occurrence) of the homologous receptor. Sequences of peptides used are indicated for each homologous receptor site. Lower-case “d”denotes the aspartic acid (Asp) residue pre-charged on the peptide synthesis resin and not a naturally occurring Asp. Peptides that resulted in no positive interactions are designated “NI”; peptides that were unable to be synthesized are designated “NS”. Rows of the heatmaps for these peptides are grayed out to indicate that our FP assay could neither confirm nor deny positive or negative interactions for these peptides.
(PDF)

Table S1 **Recombinant Src Homology 2 domains used in FP assay.**
(XLS)

Table S2 **Peptides synthesized for fluorescence polarization assay.**
(XLS)

Table S3 **Literature reports of ErbB phosphorylated tyrosines.**
(XLSX)

Table S4 **Fluorescence polarization-derived peptide-protein interaction data.**
(XLSX)

Table S5 **Sequences and significant interactions of non-phosphorylated peptides tested.**
(XLSX)

Table S6 **Summary of all original and most recently reported protein microarray-derived interactions.**
(XLSX)

Table S7 **SPR and FP cross-validation results.**
(XLSX)

Table S8 **Literature-based validation of ErbB protein-protein interactions.**
(XLSX)

Table S9 **Z’ factor values.**
(XLSX)

Table S10 **Peptide competition assay data.**
(XLSX)

Table S11 **High confidence fluorescence polarization data used to compare to protein microarray-derived interactions.**
(XLSX)

Table S12 **In-house protein microarray replicate assays.**
(XLSX)

Table S13 **Sum of binding free energies (in kcal/mole) of SH2 domains in ErbB receptors.**
(XLSX)

Table S14 **Significant enrichment or depletion of binding sites on each ErbB receptor for SH2 domains and gene ontology groups (permutation P<0.05).**
(XLSX)

Table S15 **Molecular function classes of SH2 domains.**
(XLSX)

Table S16 **Average recruitment potential for each gene ontological class as a function of relative binding free energy (in kcal/mole).**
(XLSX)

Table S17 **Peptides synthesized for in-house protein microarrays.**
(XLSX)

Methods SI A detailed description of all biochemical and mathematical analysis methods used in this study.
(DOCX)
