# Supplementary figures and images for: Comprehensive Binary Interaction Mapping of SH2 Domains via Fluorescence Polarization Reveals Novel Functional Diversification of ErbB Receptors
Source: PLoS One. 2012 Sep 4;7(9):e44471. doi: 10.1371/journal.pone.0044471 (PMC3433420; doi:10.1371/journal.pone.0044471)

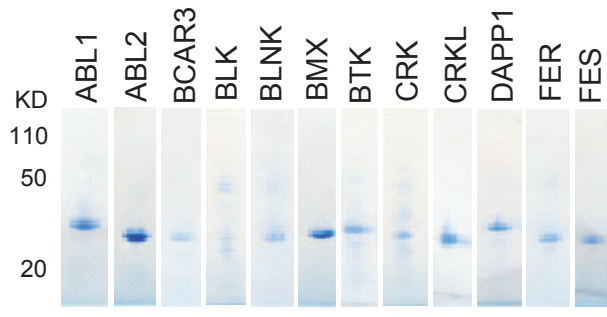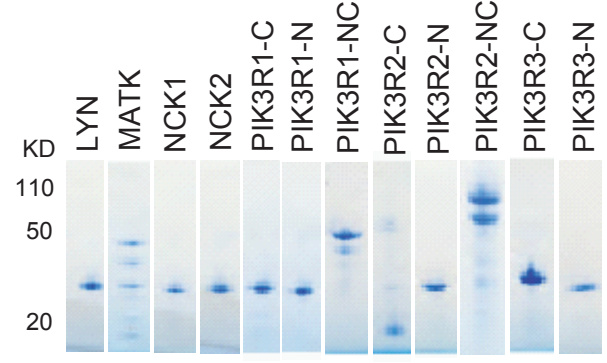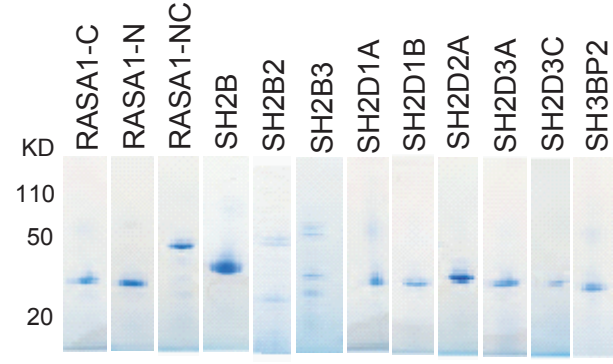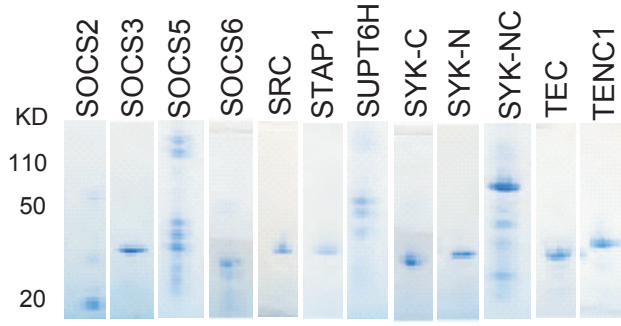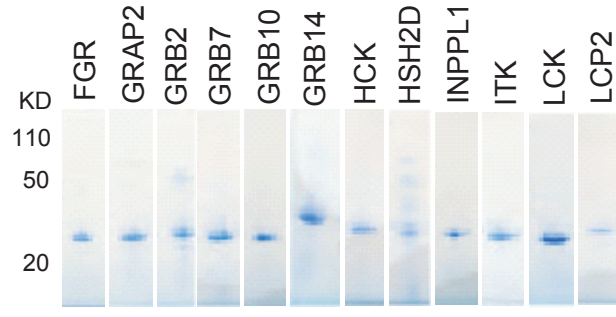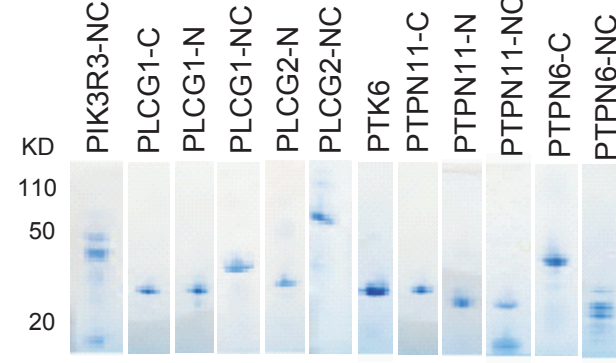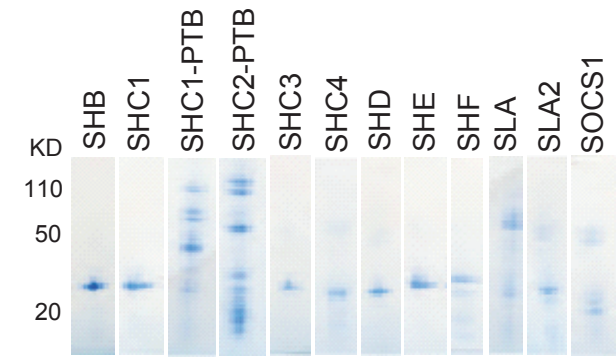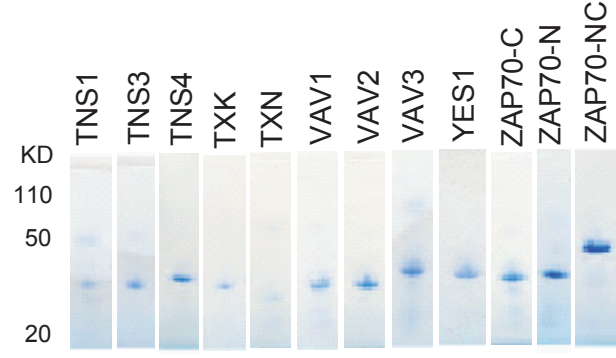

Supplement: Figure S1 — Analysis of SH2 and PTB domain purity following expression and purification. Recombinant proteins were spectrophotometrically normalized to a concentration of ∼20 µM, electrophoresed by SDS-PAGE and stained with GelCode Blue (Pierce). A representative set of all assayed proteins used in this study is displayed. (PDF) [file pone.0044471.s001.pdf]

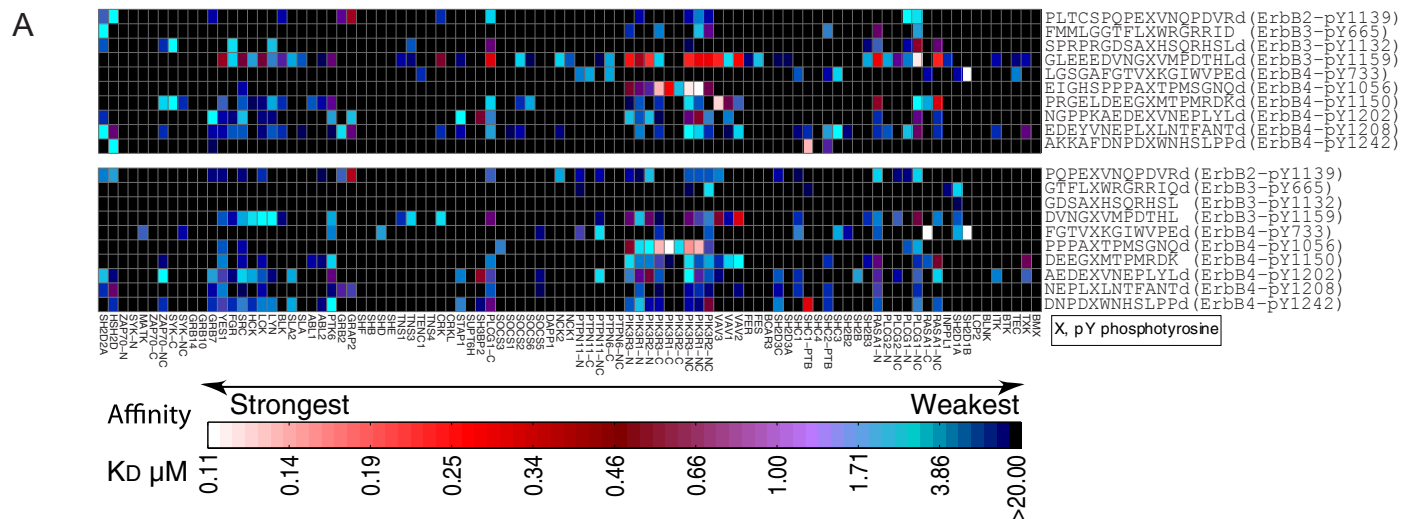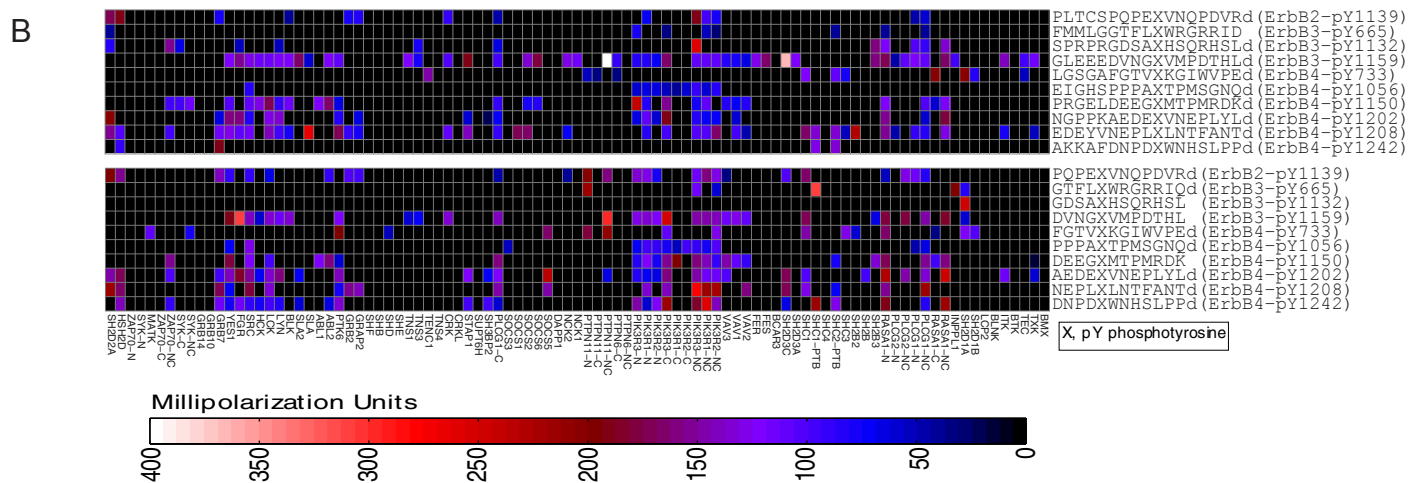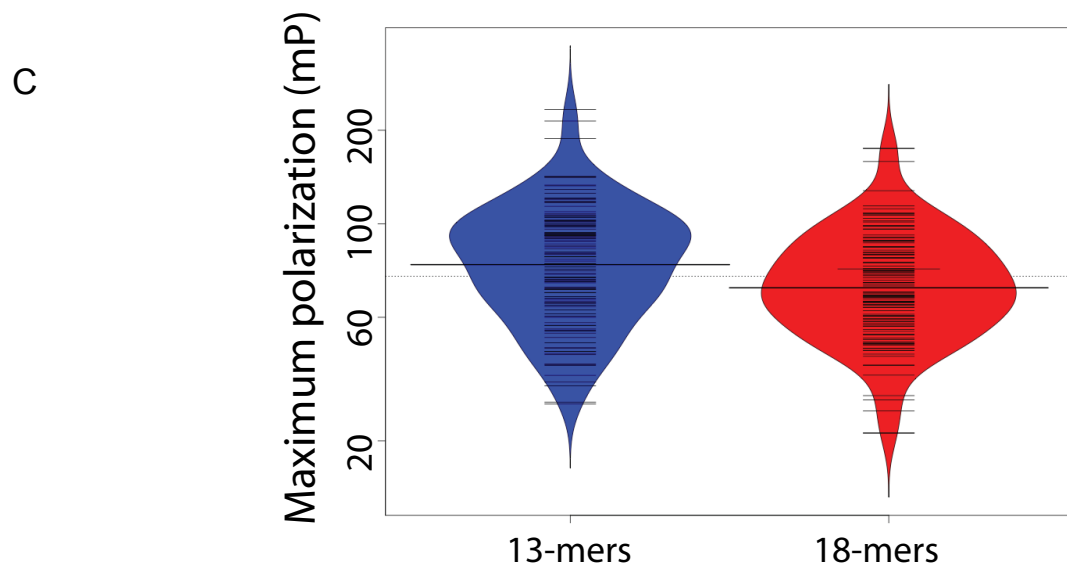

Supplement: Figure S2 — Short-form peptides produce higher polarization values than long-form peptides. (A) Heatmaps depict apparent midpoint dissociation constants (KDs) of SH2 and PTB domains with indicated 18-mer (upper panel) and 13-mer (lower panel) peptides. KDs are color coded by affinity (see scale). Lower-case “d” denotes aspartic acid (Asp) residue pre-charged on the peptide synthesis resin and not a naturally-occurring Asp. (B) Polarization values obtained by FP were fit to equation (1) and the theoretical maximum polarization values induced by SH2 and PTB domains were displayed as heat maps. (C) Side-by-side 13-mer and 18-mer bean plots of Pmax values. (PDF) [file pone.0044471.s002.pdf]

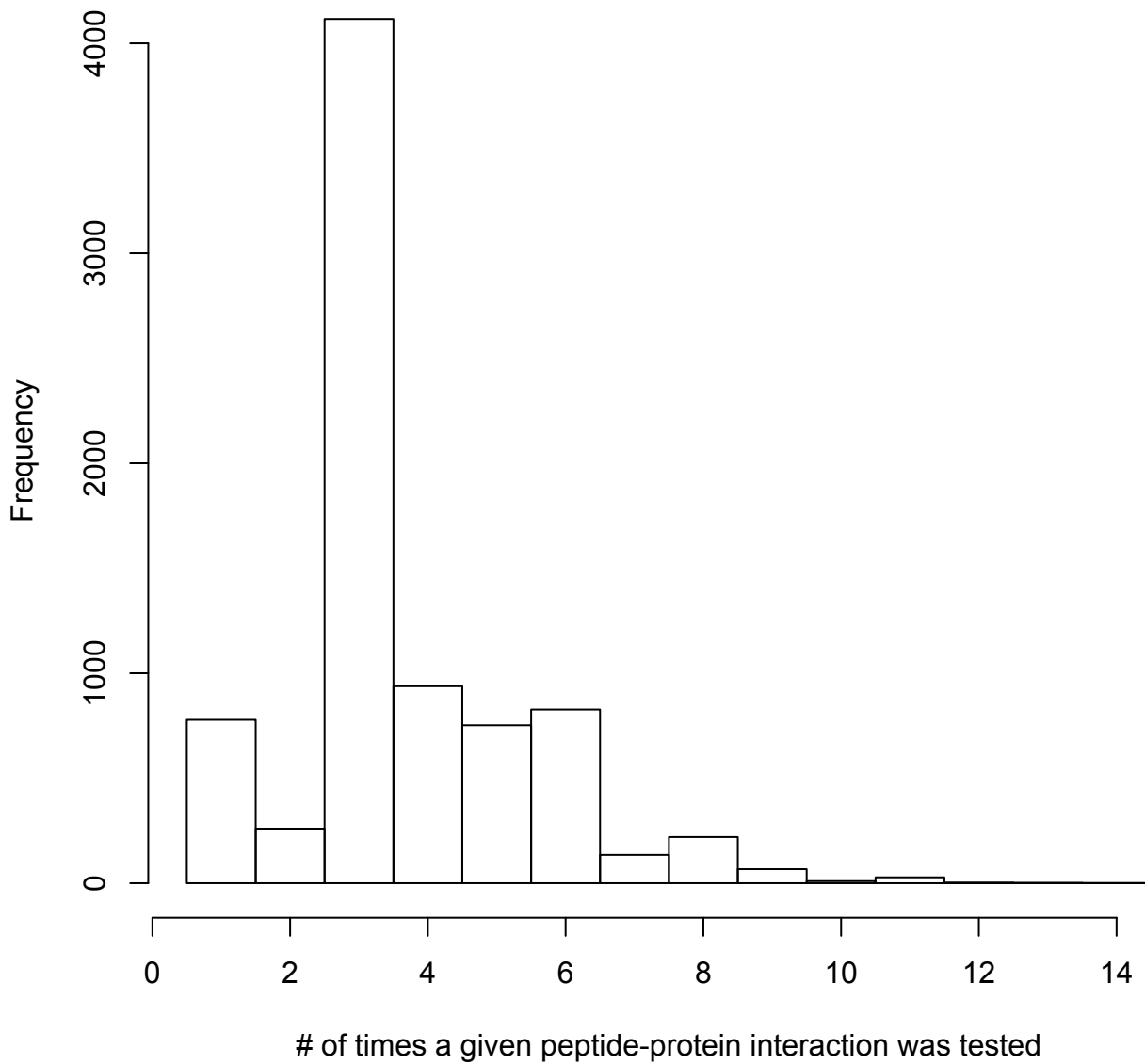

Supplement: Figure S3 — Histogram depicting how many times each peptide-protein interaction was tested across all six automated FP runs in our assay. (PDF) [file pone.0044471.s003.pdf]

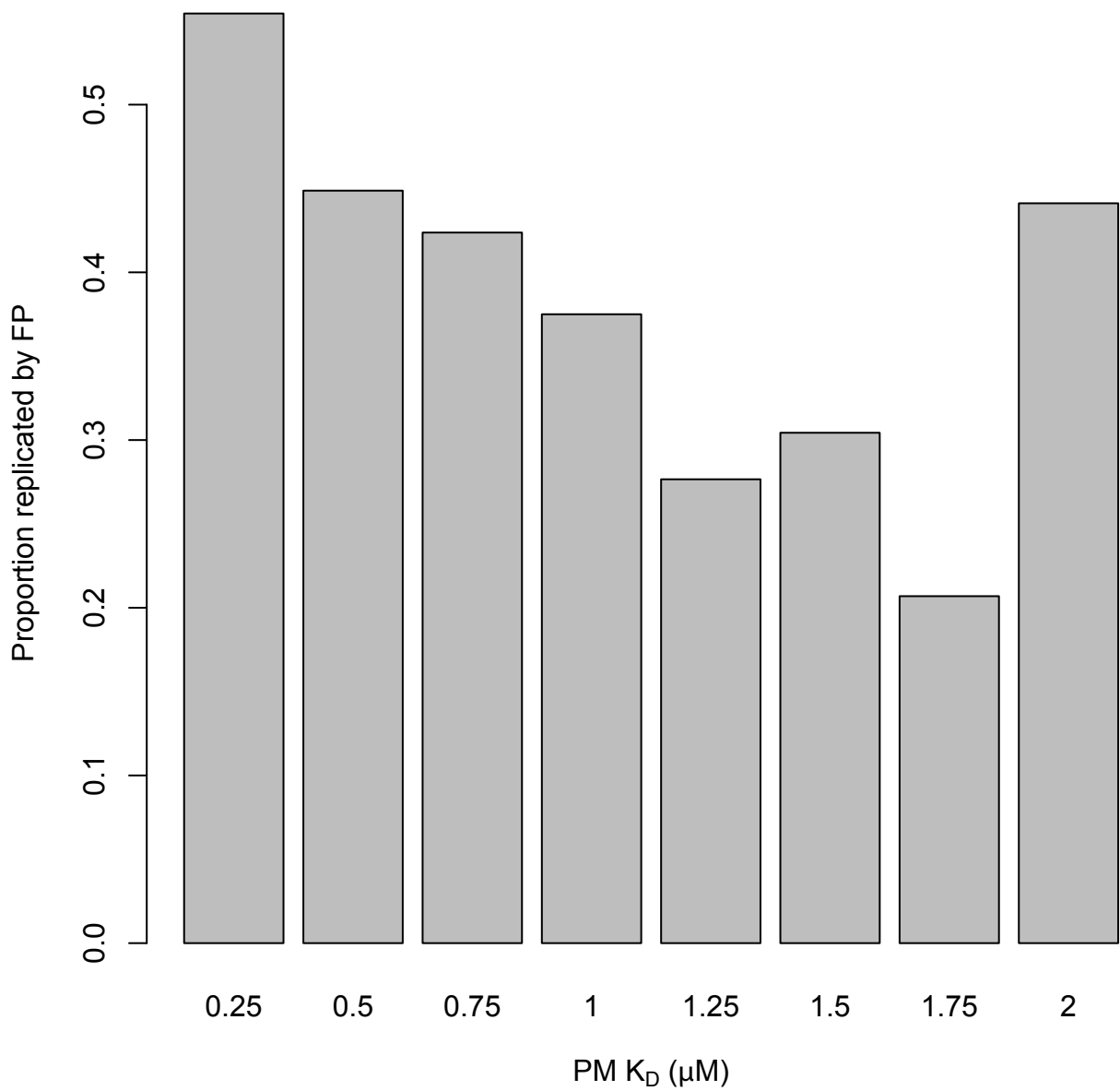

Supplement: Figure S7 — Fluorescence polarization replication probability as a function of the strength of interaction as estimated by protein microarrays. 448 interactions previously detected by protein microarrays were binned into 8 groups based on their interaction strengths and the proportion of interactions that were detected at least once by FP calculated. (PDF) [file pone.0044471.s007.pdf]

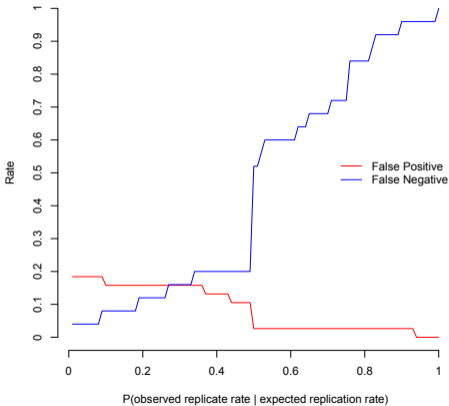

Supplement: Figure S8 — False positive rate (FPR) and false negative rate (FNR) as a function of the probability of each interaction calculated by comparing the observed versus the expected replication rate for an interaction given its strength. As expected, filtering for “high-confidence” interactions (P>0.5) gives a low FPR at the expense of greatly inflating the FNR. (PDF) [file pone.0044471.s008.pdf]

A

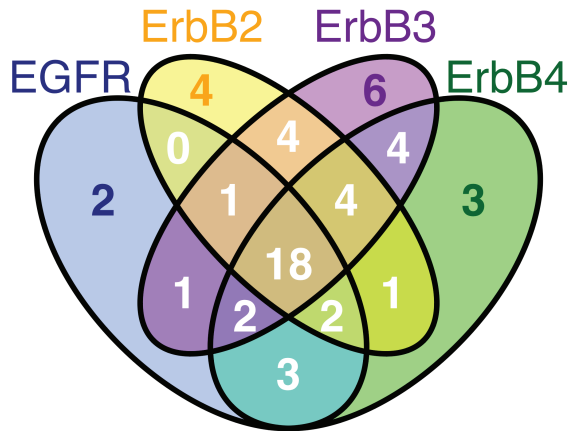

B

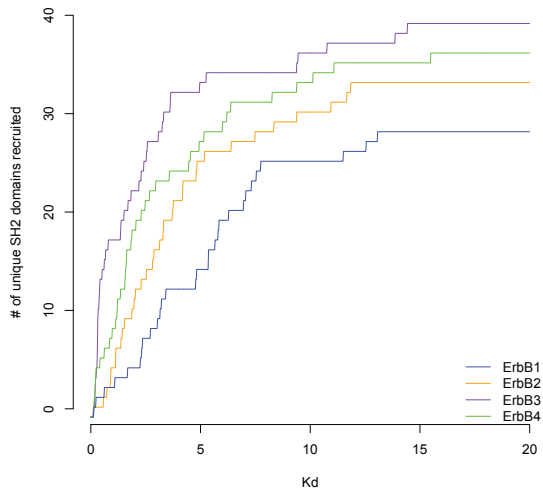

Supplement: Figure S9 — Characterization of unique and overlapping SH2 domain recruitment patterns by individual ErbB receptors for high-confidence interactions only. (A) Four-way Venn diagram depicts SH2 domain interactions shared by or exclusive to ErbB1, 2, 3, and 4. (B) SH2 recruitment potential of EGFR family members at different affinity thresholds. The total number of unique SH2 and PTB domains recruited over a range of affinity thresholds are depicted for each receptor. (PDF) [file pone.0044471.s009.pdf]

# ErbB2

# Erbb3

# ErbB4

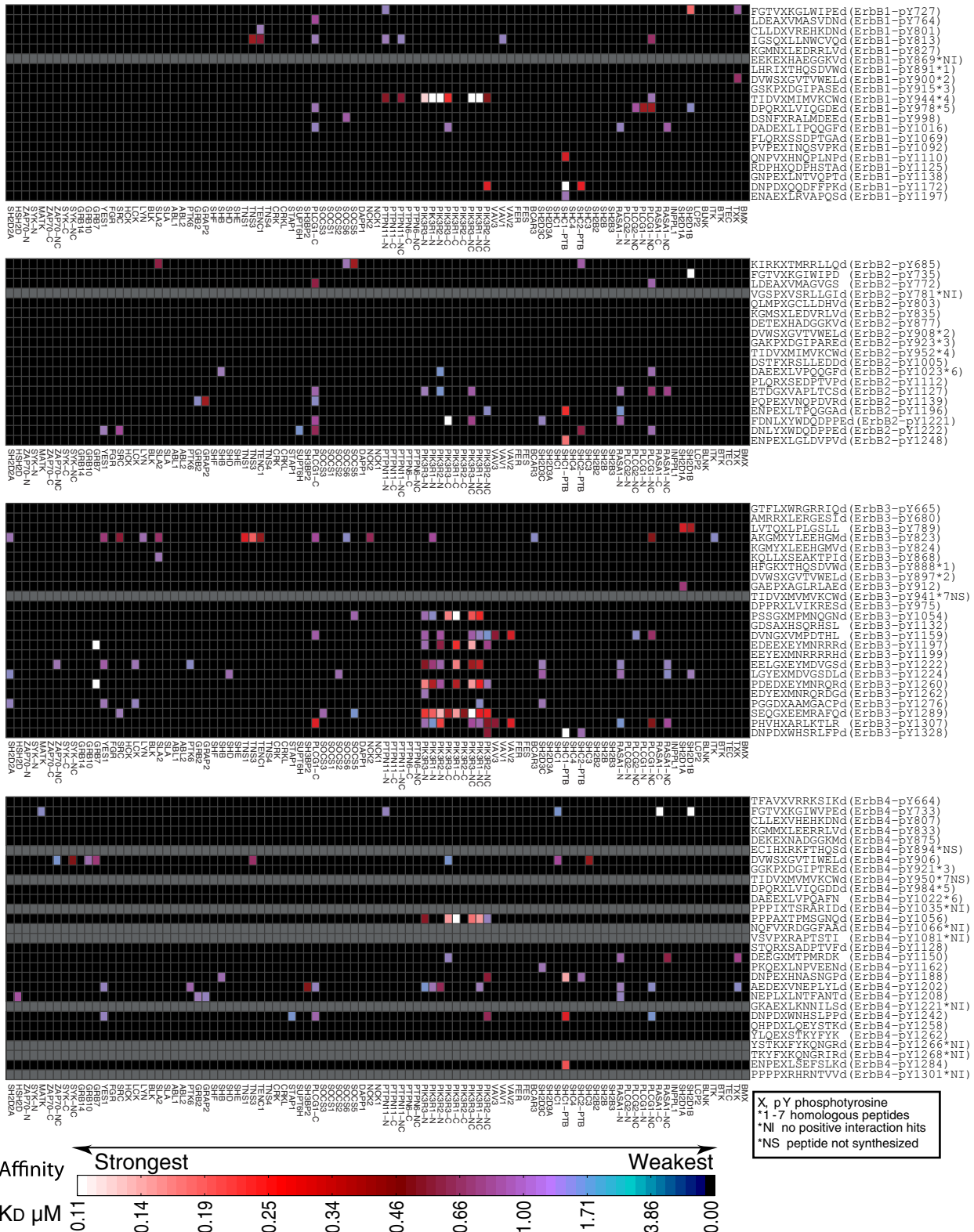

Supplement: Figure S14 — High confidence SH2 domain recruitment potential of the ErbB family as determined by high-throughput fluorescence polarization (HT-FP). Color-coded heat maps represent apparent dissociation constants (KDs) for FP interactions between SH2/PTB domains and phosphopeptides representing all potential ErbB1, ErbB2, ErbB3, and ErbB4 phosphotyrosine sites. Homologous ErbB peptides with identical amino acid residues from +1 to the +4 position relative to the phosphotyrosine (X) are indicated with an asterisk followed by the number (in order of occurrence) of the homologous receptor site. Sequences of peptides used are indicated for each homologous receptor site. Lower-case “d” denotes the aspartic acid (Asp) residue that was pre-charged on the peptide synthesis resin and not a naturally-occurring Asp. Peptides that resulted in no positive interactions are designated “NI”; peptides that were unable to be synthesized are designated “NS”. Rows of the heatmaps for these peptides are grayed out to indicate that our FP assay could neither confirm nor deny positive or negative interactions from these peptides. (PDF) [file pone.0044471.s014.pdf]

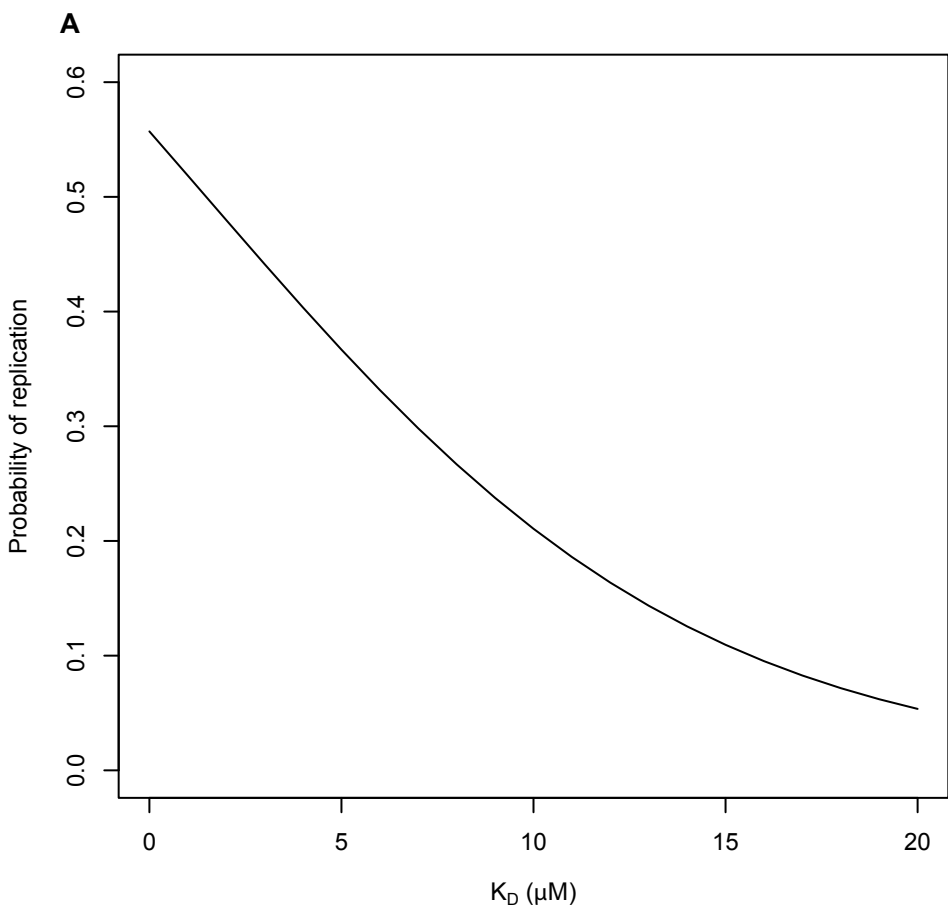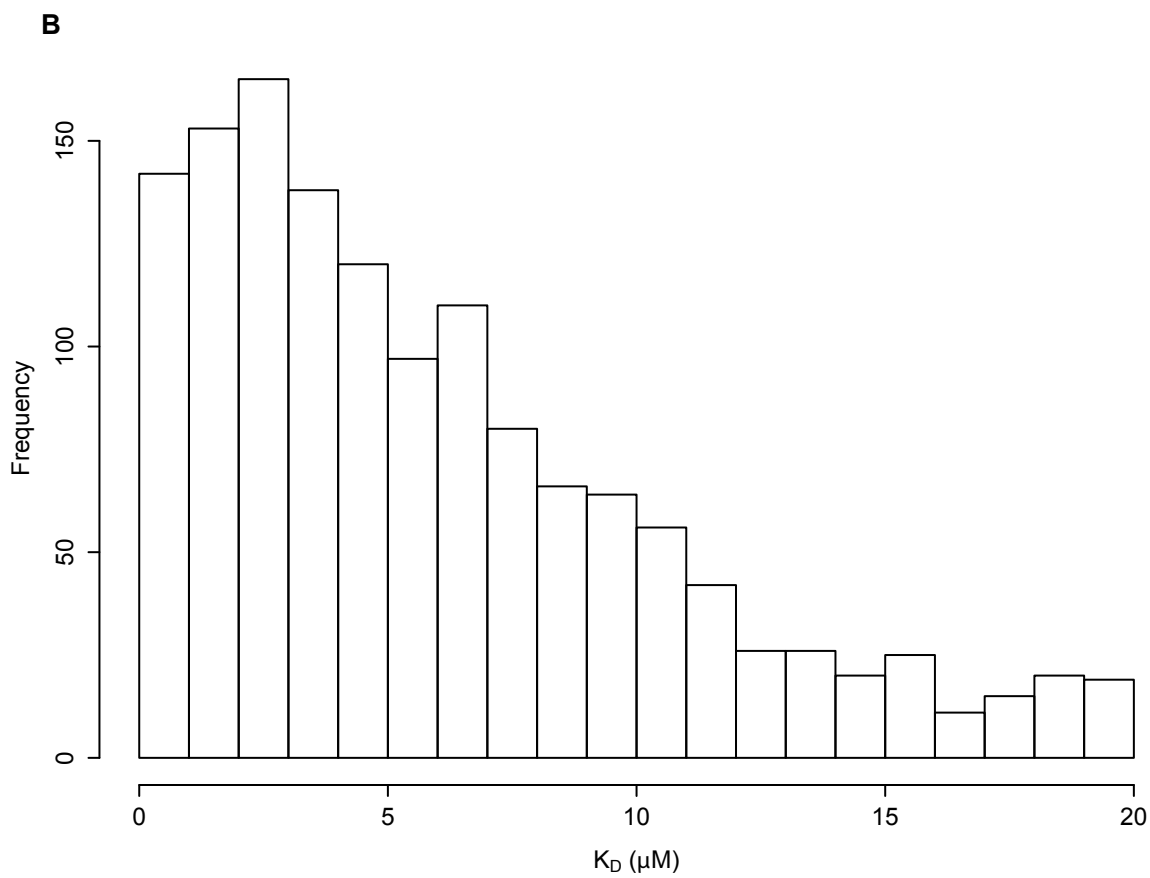

Supplement: Figure S15 — FP technical reproducibility and interaction number as a function of the strength of the interaction. (A) The probability of an interaction detected by FP being identified in subsequent runs. (B) Histogram depicts the number of interactions identified at each affinity threshold in our FP assay. (PDF) [file pone.0044471.s015.pdf]
