# Supplementary material for: Comprehensive Binary Interaction Mapping of SH2 Domains via Fluorescence Polarization Reveals Novel Functional Diversification of ErbB Receptors
Source: PLoS One. 2012 Sep 4;7(9):e44471. doi: 10.1371/journal.pone.0044471 (PMC3433420; doi:10.1371/journal.pone.0044471)
Supplement: Figure S4 — Heatmaps depicting maximum polarization values of interactions of SH2 and PTB domains with all ErbB family phosphopeptides. Polarization values obtained by FP were fit to equation (1) and the theoretical maximum polarization values induced by SH2 and PTB domains were output as heat maps. Identical peptides among the four receptors are indicated numerically. Lower-case “d” refers to the aspartic acid (Asp) residue pre-charged on the peptide synthesis resin and not a naturally-occuring Asp. Peptides that were queried but resulted in no positive interactions are designated “NI”; peptides that were unable to be synthesized are designated “NS”. Rows in the heatmaps for these peptides are greyed out to indicate that the assay could neither confirm nor deny positive or negative interactions from these peptides. (PDF) [file pone.0044471.s004.pdf]

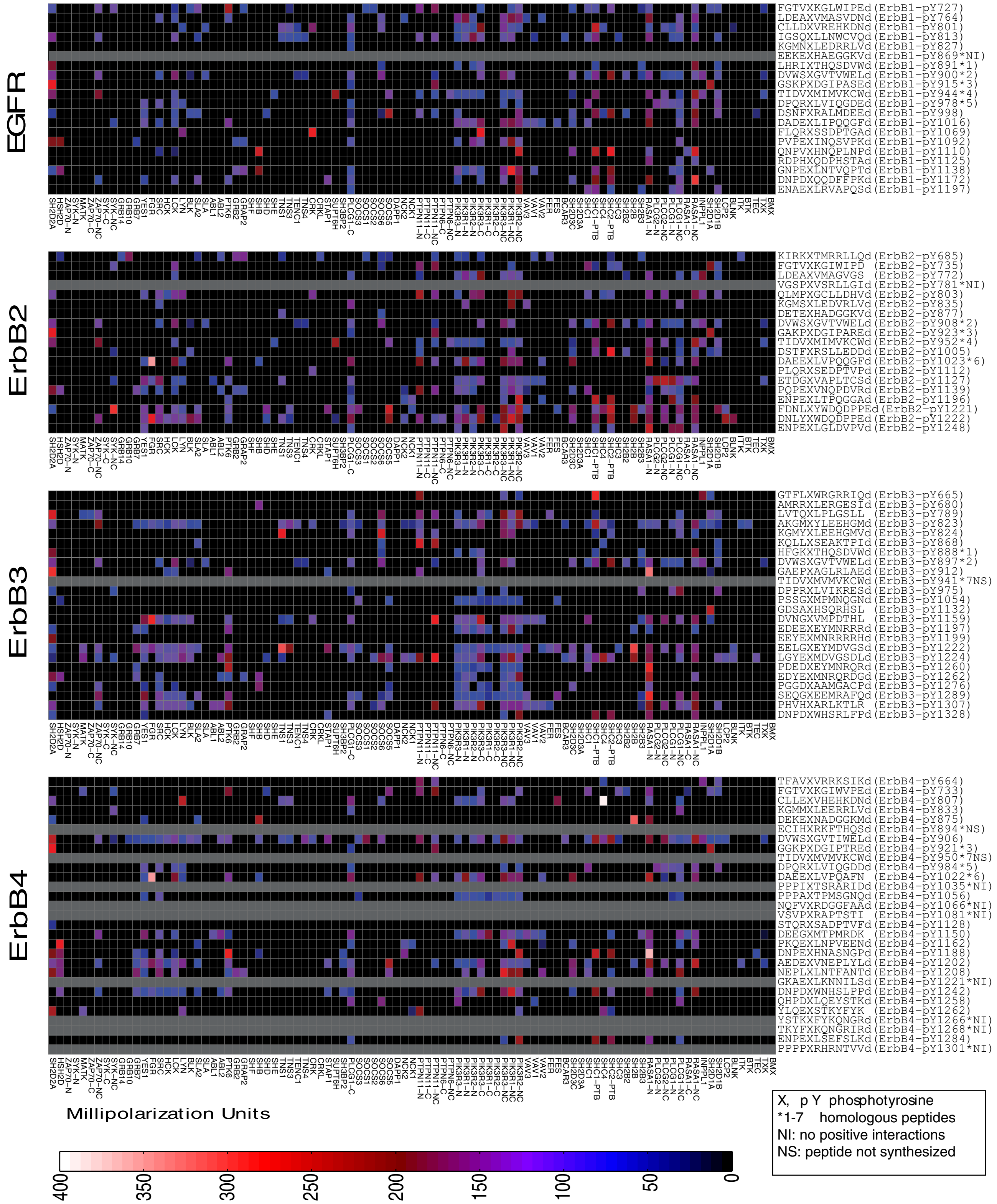
EGFR
FGTVXKGLWIPEd(ErbB1-pY727)
LDEAXVMASVDNd(ErbB1-pY764)
CLLDXVREHKDNd(ErbB1-pY801)
IGSQXLLNWCVQd(ErbB1-pY813)
KGMNXLEDRRLVd(ErbB1-pY827)
EEKEXHAEGGKVd(ErbB1-pY869*NI)
LHRIXTHQSDVWd(ErbB1-pY891*1)
DVWSXGVTVWELd(ErbB1-pY900*2)
GSKPXDGIPASEd(ErbB1-pY915*3)
TIDVXMIMVKCWd(ErbB1-pY944*4)
DPQRXLVIQGDEd(ErbB1-pY978*5)
DSNFXRALMDEEd(ErbB1-pY998)
DADEXLIPQQGFd(ErbB1-pY1016)
FLQRXSSDPTGAd(ErbB1-pY1069)
PVPEXINQSVPKd(ErbB1-pY1092)
QNPVXHNQPLNPd(ErbB1-pY1110)
RDPHXQDPHSTAd(ErbB1-pY1125)
GNPEXLNTVQPTd(ErbB1-pY1138)
DNPDXQQDFFPKd(ErbB1-pY1172)
ENAEXLRVAPQSd(ErbB1-pY1197)
SH2D2A HSH2D SYK-N MATK ZAP70-N ZAP70-C SYK-C ZAP70-NC SYK-NC GRB14 GRB10 GRB7 YES1 FGR SRC HCK LCK LYN BLK SLA2 SLA ABL1 ABL2 PTK6 GRB2 GRAP2 SHF SHD SHB SHE TNS1 TNS3 TENC1 TNS4 CRK CRKL STAP1 SUPT6H SH3BP2 PLCG1-C SOCS3 SOCS1 SOCS2 SOCS6 SOCS5 DAPP1 NCK2 NCK1 PTPN11-N PTPN11-C PTPN11-NC PTPN6-C PTPN6-NC PIK3R3-N PIK3R1-N PIK3R2-N PIK3R3-C PIK3R1-C PIK3R2-C PIK3R3-NC PIK3R1-NC PIK3R2-NC VAV3 VAV1 VAV2 FER FES BCAR3 SH2D3C SH2D3A SHC1 SHC1-PTB SHC4 SHC2-PTB SHC3 SH2B2 SH2B SH2B3 RASA1-N PLCG2-N PLCG2-NC PLCG1-N PLCG1-NC RASA1-C RASA1-NC INPPL1 SH2D1A SH2D1B LCP2 BLNK ITK BTK TEC TXK BMX
ErbB2
KIRKXTMRRLLQd(ErbB2-pY685)
FGTVXKGIWIPD (ErbB2-pY735)
LDEAXVMAGVGS (ErbB2-pY772)
VGSPXVSRLLGId(ErbB2-pY781*NI)
QLMPXGCLLDHVd(ErbB2-pY803)
KGMSXLEDVRLVd(ErbB2-pY835)
DETEXHADGGKVd(ErbB2-pY877)
DVWSXGVTVWELd(ErbB2-pY908*2)
GAKPXDGIPAREd(ErbB2-pY923*3)
TIDVXMIMVKCWd(ErbB2-pY952*4)
DSTFXRSLLEDDd(ErbB2-pY1005)
DAEEXLVPQQGFd(ErbB2-pY1023*6)
PLQRXSEDPTVPd(ErbB2-pY1112)
ETDGXVAPLTCSd(ErbB2-pY1127)
PQPEXVNQPDVRd(ErbB2-pY1139)
ENPEXLTPQGGAd(ErbB2-pY1196)
FDNLXYWDQDPPEd(ErbB2-pY1221)
DNLYXWDQDPPEd(ErbB2-pY1222)
ENPEXLGLDVPVd(ErbB2-pY1248)
ErbB3
GTFLXWRGRRIQd(ErbB3-pY665)
AMRRXLERGESId(ErbB3-pY680)
LVTQXLPLGSLL (ErbB3-pY789)
AKGMXYLEEHGMd(ErbB3-pY823)
KGMYXLEEHGMVd(ErbB3-pY824)
KQLLXSEAKTPId(ErbB3-pY868)
HFGKXTHQSDVWd(ErbB3-pY888*1)
DVWSXGVTVWELd(ErbB3-pY897*2)
GAEPXAGLRLAEd(ErbB3-pY912)
TIDVXMVMVKCWd(ErbB3-pY941*7NS)
DPPRXLVIKRESd(ErbB3-pY975)
PSSGXMPMNQGNd(ErbB3-pY1054)
GDSAXHSQRHSL (ErbB3-pY1132)
DVNGXVMPDTHL (ErbB3-pY1159)
EDEEXEYMNRRRd(ErbB3-pY1197)
EEYEXMNRRRRHd(ErbB3-pY1199)
EELGXEYMDVGSd(ErbB3-pY1222)
LGYEXMDVGSDLd(ErbB3-pY1224)
PDEDXEYMNRQRd(ErbB3-pY1260)
EDYEXMNRQRDGd(ErbB3-pY1262)
PGGDXAAMGACPd(ErbB3-pY1276)
SEQGXEEMRAFQd(ErbB3-pY1289)
PHVHXARLKTLR (ErbB3-pY1307)
DNPDXWHSRLFPd(ErbB3-pY1328)
ErbB4
TFAVXVRRKSIKd(ErbB4-pY664)
FGTVXKGIWVPEd(ErbB4-pY733)
CLLEXVHEHKDNd(ErbB4-pY807)
KGMMXLEERRLVd(ErbB4-pY833)
DEKEXNADGGKMd(ErbB4-pY875)
ECIHXRKFTHQSd(ErbB4-pY894*NS)
DVWSXGVTIWELd(ErbB4-pY906)
GGKPXDGIPTREd(ErbB4-pY921*3)
TIDVXMVMVKCWd(ErbB4-pY950*7NS)
DPQRXLVIQGDDd(ErbB4-pY984*5)
DAEEXLVPQAFN (ErbB4-pY1022*6)
PPPIXTSRARIDd(ErbB4-pY1035*NI)
PPAXTPMSGNQd(ErbB4-pY1056)
NQFVXRDGGFAAd(ErbB4-pY1066*NI)
VSVPXRAPTSTI (ErbB4-pY1081*NI)
STQRXSADPTVFd(ErbB4-pY1128)
DEEGXMTPMRDK (ErbB4-pY1150)
PKQEXLNPVEENd(ErbB4-pY1162)
DNPEXHNASNGPd(ErbB4-pY1188)
AEDEXVNEPLYLd(ErbB4-pY1202)
NEPLXLNTFANTd(ErbB4-pY1208)
GKAEXLKNNILSd(ErbB4-pY1221*NI)
DNPDXWNHSLPPd(ErbB4-pY1242)
QHPDXLQEYSTKd(ErbB4-pY1258)
YLQEXSTKYFYK (ErbB4-pY1262)
YSTKXFYKQNGRd(ErbB4-pY1266*NI)
TKYFXKQNGRIRd(ErbB4-pY1268*NI)
ENPEXLSEFSLKd(ErbB4-pY1284)
PPPPXRHRNTVVd(ErbB4-pY1301*NI)
Millipolarization Units
400 350 300 250 200 150 100 50 0
X, pY phosphotyrosine
*1-7 homologous peptides
NI: no positive interactions
NS: peptide not synthesized
