# Supplementary material for: Comprehensive Binary Interaction Mapping of SH2 Domains via Fluorescence Polarization Reveals Novel Functional Diversification of ErbB Receptors
Source: PLoS One. 2012 Sep 4;7(9):e44471. doi: 10.1371/journal.pone.0044471 (PMC3433420; doi:10.1371/journal.pone.0044471)
Supplement: Figure S5 — SH2 domain recruitment potential of the ErbB family as previously determined by protein microarrays. Color-coded heat maps (see legend) represent apparent dissociation constants (KDs) for protein microarray interactions between SH2/PTB domains and phosphopeptides representing potential ErbB1, ErbB2, ErbB3 and ErbB4 phosphotyrosine sites. Binding strength is color-coded as indicated on the legend. Homologous peptides with identical amino acid sequences at the +1 to the +4 position relative to the phosphotyrosine (X) are marked with an asterisk followed by the number of the homologous receptor with sequences indicated. Lower-case “d” denotes the aspartic acid (Asp) residue pre-charged on the peptide synthesis resin and not a naturally-occurring Asp. Rows of the heatmaps for peptides that have no previously reported protein microarray interactions are grayed out to indicate that no experiments were performed to confirm or deny positive or negative interactions from these peptides. (PDF) [file pone.0044471.s005.pdf]

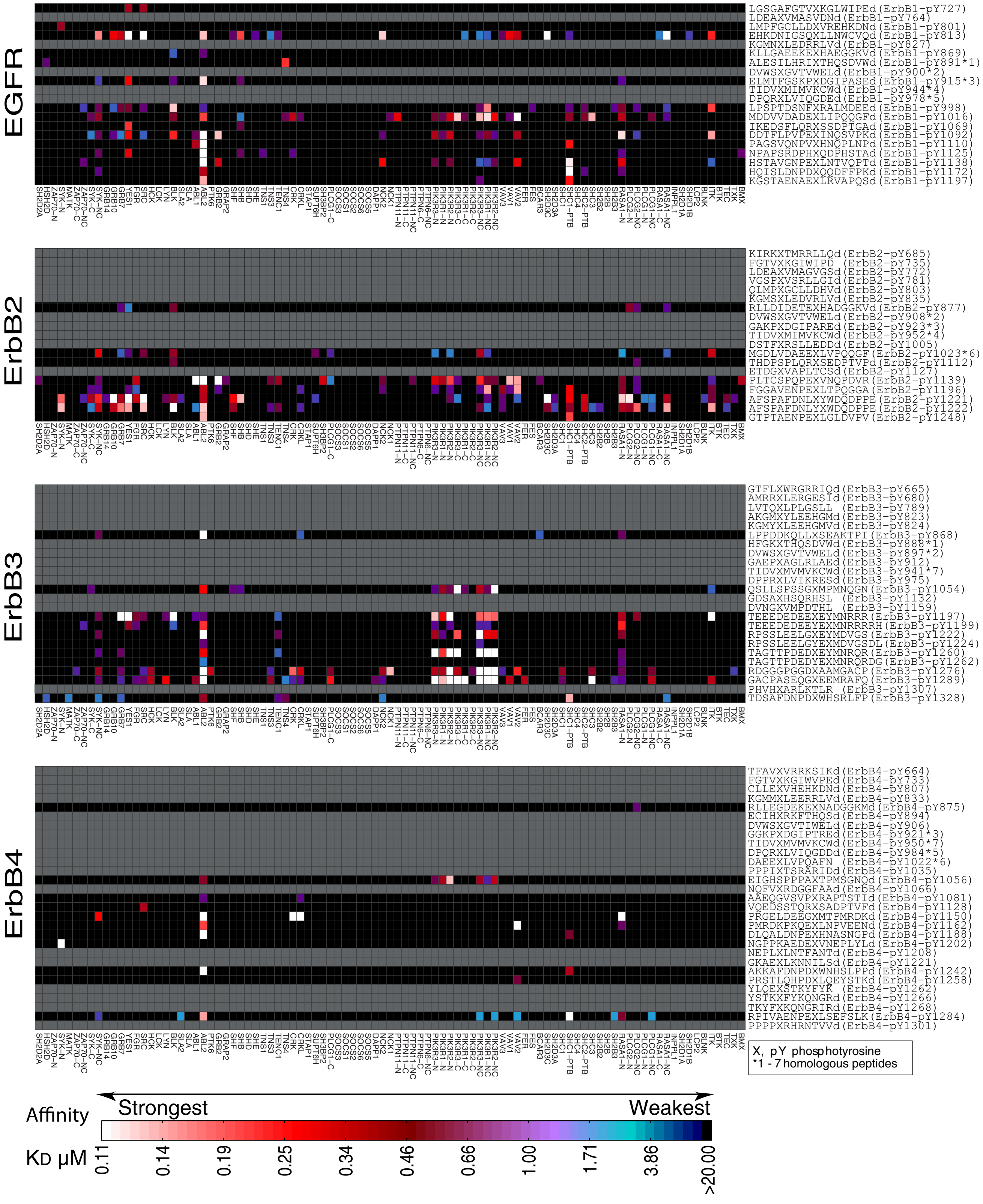

EGFR
LGSGAFGTVXKGLWIPEd (ErbB1-pY727)
LDEAXVMASVDNd (ErbB1-pY764)
LMPFGCLLDXVREHKDNd (ErbB1-pY801)
EHKDNIGSQXLLNWCVQd (ErbB1-pY813)
KGMNXLEDRRLVd (ErbB1-pY827)
KLLGAEEKEXHAEGGKVd (ErbB1-pY869)
ALESILHRIXTHQSDVWd (ErbB1-pY891*1)
DVWSXGVTVWELd (ErbB1-pY900*2)
ELMTFGSKPXDGIPASEd (ErbB1-pY915*3)
TIDVXMIMVKCWd (ErbB1-pY944*4)
DPQRXLVIQGDEd (ErbB1-pY978*5)
LPSPTDSNFXRALMDEEd (ErbB1-pY998)
MDDVVDADEXLIPQQGFd (ErbB1-pY1016)
IKEDSFLQRXSSDPTGAd (ErbB1-pY1069)
DDTFLPVPEXINQSVPKd (ErbB1-pY1092)
PAGSVQNPVXHNQPLNPd (ErbB1-pY1110)
NPAPSRDPHXQDPHSTAd (ErbB1-pY1125)
HSTAVGNPEXLNTVQPTd (ErbB1-pY1138)
HQISLDNPDXQQDFFPKd (ErbB1-pY1172)
KGSTAENAEXLRVAPQSd (ErbB1-pY1197)
SH2D2A HSH2D ZAP70-N MATK ZAP70-C ZAP70-NC SYK-C SYK-NC GRB14 GRB10 GRB7 YES1 FGR SRC HCK LCK LYN BLK SLA2 SLA ABL1 ABL2 PTK6 GRB2 GRAP2 SHF SHB SHD SHE TNS1 TNS3 TENC1 TNS4 CRK CRKL SUPT6H SH3BP2 PLCG1-C SOCS3 SOCS2 SOCS6 SOCS5 DAPP1 NCK2 NCK1 PTPN11-N PTPN11-C PTPN11-NC PTPN6-C PTPN6-NC PIK3R3-N PIK3R1-N PIK3R2-N PIK3R3-C PIK3R2-C PIK3R1-C PIK3R3-NC PIK3R1-NC PIK3R2-NC VAV3 VAV1 VAV2 FER FES BCAR3 SH2D3C SH2D3A SHC1 SHC1-PTB SHC4 SHC2-PTB SHC3 SH2B2 SH2B3 RASA1-N PLCG2-N PLCG2-NC PLCG1-N PLCG1-NC RASA1-C RASA1-NC INPPL1 SH2D1A SH2D1B BLNK ITK BTK TEC TXK BMX
ErbB2
KIRKXTMRRLLQd (ErbB2-pY685)
FGTVXKGIWIPD (ErbB2-pY735)
LDEAXVMAGVGSd (ErbB2-pY772)
VGSPXVSRLLGId (ErbB2-pY781)
QLMPXGCLLDHVd (ErbB2-pY803)
KGMSXLEDVRLVd (ErbB2-pY835)
RLLDIDETEXHADGGKVd (ErbB2-pY877)
DVWSXGVTVWELd (ErbB2-pY908*2)
GAKPXDGIPAREd (ErbB2-pY923*3)
TIDVXMIMVKCWd (ErbB2-pY952*4)
DSTFXRSLLEDDd (ErbB2-pY1005)
MGDLVDAEEXLVPQQGF (ErbB2-pY1023*6)
THDPSPLQRXSEDPTVPd (ErbB2-pY1112)
ETDGXVAPLTCSd (ErbB2-pY1127)
PLTCSPQPEXVNQPDVR (ErbB2-pY1139)
FGGAVENPEXLTPQGGA (ErbB2-pY1196)
AFSPAFDNLXYWDQDPPE (ErbB2-pY1221)
AFSPAFDNLYXWDQDPPE (ErbB2-pY1222)
GTPTAENPEXLGLDVPV (ErbB2-pY1248)
ErbB3
GTFLXWRGRRIQd (ErbB3-pY665)
AMRRXLERGESId (ErbB3-pY680)
LVTQXLPLGSLL (ErbB3-pY789)
AKGMXYLEEHGMd (ErbB3-pY823)
AKGMYXLEEHGMVd (ErbB3-pY824)
LPPDDKQLLXSEAKTPI (ErbB3-pY868)
HFGKXTHQSDVWd (ErbB3-pY888*1)
DVWSXGVTVWELd (ErbB3-pY897*2)
GAEPXAGLRLAEd (ErbB3-pY912)
TIDVXMVMVKCWd (ErbB3-pY941*7)
DPPRXLVIKRESd (ErbB3-pY975)
QSLLSPSSGXMPMNQGN (ErbB3-pY1054)
GDSAXHSQRHSL (ErbB3-pY1132)
DVNGXVMPDTHL (ErbB3-pY1159)
TEEEDEDEEXEYMNRRR (ErbB3-pY1197)
TEEEDEDEEYEXMNRRRRH (ErbB3-pY1199)
RPSSLEELGXEYMDVGS (ErbB3-pY1222)
RPSSLEELGYEXMDVGSDL (ErbB3-pY1224)
TAGTTPDEDXEYMNRQR (ErbB3-pY1260)
TAGTTPDEDYEXMNRQRDG (ErbB3-pY1262)
RDGGGPGGDXAAMGACP (ErbB3-pY1276)
GACPASEQGXEEMRAFQ (ErbB3-pY1289)
PHVHXARLKTLR (ErbB3-pY1307)
TDSAFDNPDXWHSRLFP (ErbB3-pY1328)
ErbB4
TFAVXVRRKSIKd (ErbB4-pY664)
FGTVXKGIWVPEd (ErbB4-pY733)
CLLEXVHEHKDNd (ErbB4-pY807)
KGMMXLEERRLVd (ErbB4-pY833)
RLLEGDEKEXNADGGKMd (ErbB4-pY875)
ECIHXRKFTHQSd (ErbB4-pY894)
DVWSXGVTIWELd (ErbB4-pY906)
GGKPXDGIPTREd (ErbB4-pY921*3)
TIDVXMVMVKCWd (ErbB4-pY950*7)
DPQRXLVIQGDDd (ErbB4-pY984*5)
DAEEXLVPQAFN (ErbB4-pY1022*6)
PPIXTSRARIDd (ErbB4-pY1035)
EIGHSPPPAXTPMSGNQd (ErbB4-pY1056)
NQFVXRDGGFAAd (ErbB4-pY1066)
AAEQGVSVPXRAPTSTId (ErbB4-pY1081)
VQEDSSTQRXSADPTVFd (ErbB4-pY1128)
PRGELDEEGXMTPMRDKd (ErbB4-pY1150)
PMRDKPKQEXLNPVEENd (ErbB4-pY1162)
DLQALDNPEXHNASNGPd (ErbB4-pY1188)
NGPPKAEDEXVNEPLYLd (ErbB4-pY1202)
NEPLXLNTFANTd (ErbB4-pY1208)
GKAEXLKNNILSd (ErbB4-pY1221)
AKKAFDNPDXWNHSLPPd (ErbB4-pY1242)
PRSTLQHPDXLQEYSTKd (ErbB4-pY1258)
YLQEXSTKYFYK (ErbB4-pY1262)
YSTKXFYKQNGRd (ErbB4-pY1266)
TKYFXKQNGRIRd (ErbB4-pY1268)
RPIVAENPEXLSEFSLK (ErbB4-pY1284)
PPPPXRHRNTVVd (ErbB4-pY1301)
X, pY phosphotyrosine
*1 - 7 homologous peptides
Affinity
Strongest
Weakest
KD μM
0.11
0.14
0.19
0.25
0.34
0.46
0.66
1.00
1.71
3.86
>20.00
