# Supplementary material for: Comprehensive Binary Interaction Mapping of SH2 Domains via Fluorescence Polarization Reveals Novel Functional Diversification of ErbB Receptors
Source: PLoS One. 2012 Sep 4;7(9):e44471. doi: 10.1371/journal.pone.0044471 (PMC3433420; doi:10.1371/journal.pone.0044471)
Supplement: Figure S6 — Overlap between protein microarrays and fluorescence polarization approaches. Interactions detected by protein microarrays (PMs) in previous studies are plotted with interactions detected by the fluorescence polarization (FP) assay in this study. Interactions detected by PMs alone are colored green. Interactions detected by FP alone are colored red. Interactions detected by both platforms are colored in yellow. Homologous peptides with identical amino acid sequences at the +1 to the +4 position relative to the phosphotyrosine (X) are marked with an asterisk followed by the number of the homologous receptor with sequences indicated. Lower-case “d” denotes the aspartic acid (Asp) residue pre-charged on the peptide synthesis resin and not a naturally-occurring Asp. Peptides that were unable to be synthesized (NS) and those that were queried but resulted in no positive interactions (NI) are indicated. Rows of the heatmaps for these peptides are grayed out to indicate that neither FP nor PM could experimentally confirm or deny positive or negative interactions from these peptides. (PDF) [file pone.0044471.s006.pdf]

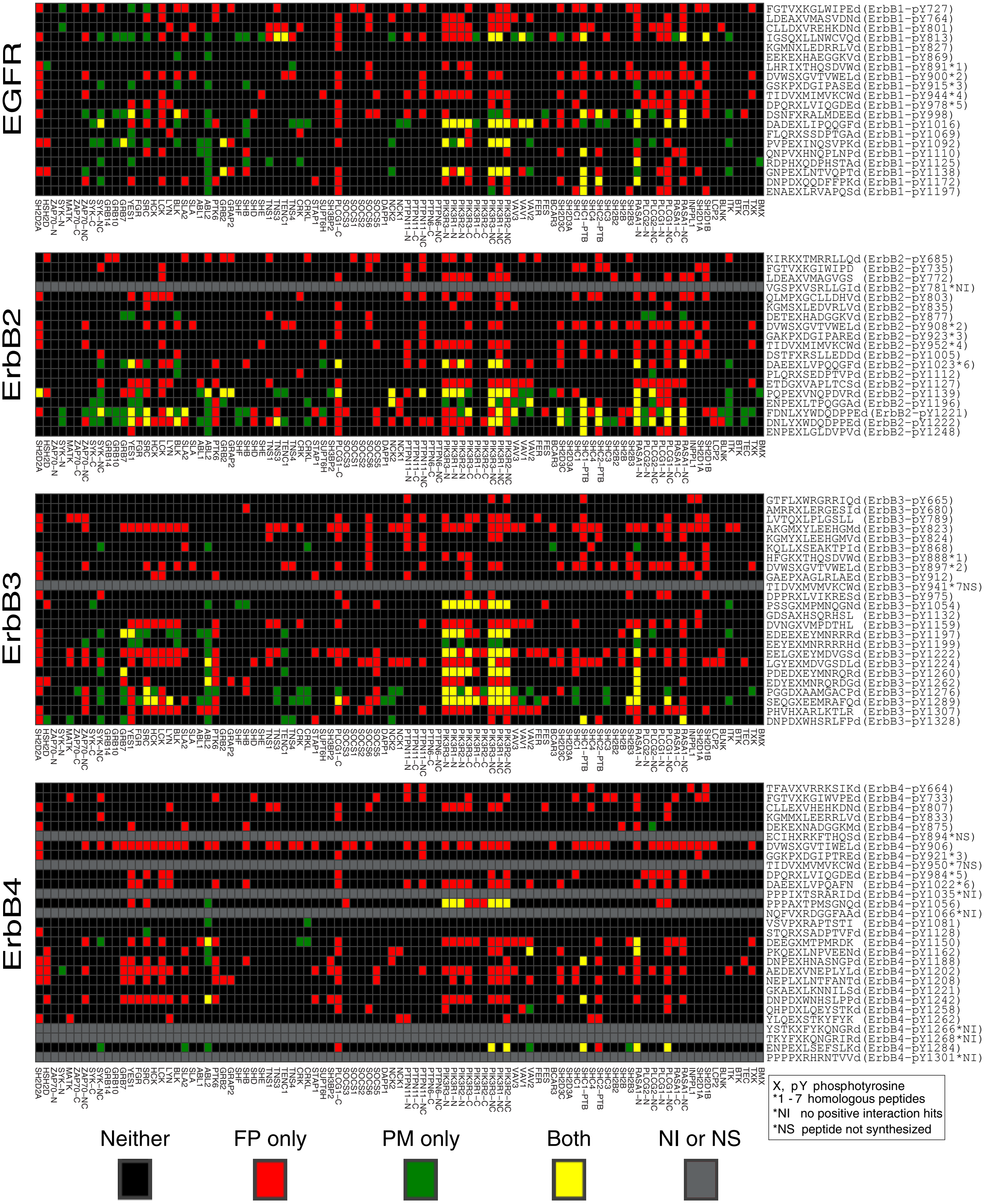
EGFR
FGTVXKGLWIPEd(ErbB1-pY727)
LDEAXVMASVDNd(ErbB1-pY764)
CLLDXVREHKDNd(ErbB1-pY801)
IGSQXLLNWCVQd(ErbB1-pY813)
KGMNXLEDRRLVd(ErbB1-pY827)
EEKEXHAEGGKVd(ErbB1-pY869)
LHRIXTHQSDVWd(ErbB1-pY891*1)
DVWSXGVTVWELd(ErbB1-pY900*2)
GSKPXDGIPASEd(ErbB1-pY915*3)
TIDVXMIMVKCWd(ErbB1-pY944*4)
DPQRXLVIQGDEd(ErbB1-pY978*5)
DSNFXRALMDEEd(ErbB1-pY998)
DADEXLIPQQGFd(ErbB1-pY1016)
FLQRXSSDPTGAd(ErbB1-pY1069)
PVPEXINQSVPKd(ErbB1-pY1092)
QNPVXHNQPLNPd(ErbB1-pY1110)
RDPHXQDPHSTAd(ErbB1-pY1125)
GNPEXLNTVQPTd(ErbB1-pY1138)
DNPDXQQDFFPKd(ErbB1-pY1172)
ENAEXLRVAPQSd(ErbB1-pY1197)
SH2D2A HSH2D SYK-N ZAP70-N MATK ZAP70-C ZAP70-NC SYK-C SYK-NC GRB14 GRB10 GRB7 YES1 FGR SRC HCK LCK LYN BLK SLA2 SLA ABL1 ABL2 PTK6 GRB2 GRAP2 SHF SHB SHD SHE TNS1 TNS3 TENC1 TNS4 CRK CRKL STAP1 SUPT6H SH3BP2 PLCG1-C SOCS3 SOCS1 SOCS2 SOCS6 SOCS5 DAPP1 NCK1 NCK2 PTPN11-N PTPN11-C PTPN11-NC PTPN6-C PTPN6-NC PIK3R3-N PIK3R1-N PIK3R2-N PIK3R3-C PIK3R1-C PIK3R2-C PIK3R3-NC PIK3R1-NC PIK3R2-NC VAV3 VAV1 VAV2 FER FES BCAR3 SH2D3C SH2D3A SHC1 SHC1-PTB SHC4 SHC2-PTB SHC3 SH2B2 SH2B SH2B3 RASA1-N PLCG2-N PLCG2-NC PLCG1-N PLCG1-NC RASA1-C RASA1-NC INPPL1 SH2D1A SH2D1B LCP2 BLNK ITK BTK TEC TXK BMX
ErbB2
KIRKXTMRRLLQd(ErbB2-pY685)
FGTVXKGIWIPD (ErbB2-pY735)
LDEAXVMAGVGS (ErbB2-pY772)
VGSPXVSRLLGId(ErbB2-pY781*NI)
QLMPXGCLLDHVd(ErbB2-pY803)
KGMSXLEDVRLVd(ErbB2-pY835)
DETEXHADGGKVd(ErbB2-pY877)
DVWSXGVTVWELd(ErbB2-pY908*2)
GAKPXDGIPAREd(ErbB2-pY923*3)
TIDVXMIMVKCWd(ErbB2-pY952*4)
DSTFXRSLLEDDd(ErbB2-pY1005)
DAEEXLVPQQGFd(ErbB2-pY1023*6)
PLQRXSEDPTVPd(ErbB2-pY1112)
ETDGXVAPLTCSd(ErbB2-pY1127)
PQPEXVNQPDVRd(ErbB2-pY1139)
ENPEXLTPQGGAd(ErbB2-pY1196)
FDNLXYWDQDPPEd(ErbB2-pY1221)
DNLYXWDQDPPEd(ErbB2-pY1222)
ENPEXLGLDVPVd(ErbB2-pY1248)
ErbB3
GTFLXWRGRRIQd(ErbB3-pY665)
AMRRXLERGESId(ErbB3-pY680)
LVTQXLPLGSLL (ErbB3-pY789)
AKGMXYLEEHGMd(ErbB3-pY823)
KGMYXLEEHGMVd(ErbB3-pY824)
KQLLXSEAKTPId(ErbB3-pY868)
HFGKXTHQSDVWd(ErbB3-pY888*1)
DVWSXGVTVWELd(ErbB3-pY897*2)
GAEPXAGLRLAEd(ErbB3-pY912)
TIDVXMVMVKCWd(ErbB3-pY941*7NS)
DPPRXLVIKRESd(ErbB3-pY975)
PSSGXMPMNQGNd(ErbB3-pY1054)
GDSAXHSQRHSL (ErbB3-pY1132)
DVNGXVMPDTHL (ErbB3-pY1159)
EDEEXEYMNRRRd(ErbB3-pY1197)
EEYEXMNRRRRHd(ErbB3-pY1199)
EELGXEYMDVGSd(ErbB3-pY1222)
LGYEXMDVGSDLd(ErbB3-pY1224)
PDEDXEYMNRQRd(ErbB3-pY1260)
EDYEXMNRQRDGd(ErbB3-pY1262)
PGGDXAAMGACPd(ErbB3-pY1276)
SEQGXEEMRAFQd(ErbB3-pY1289)
PHVHXARLKTLR (ErbB3-pY1307)
DNPDXWHSRLFPd(ErbB3-pY1328)
ErbB4
TFAVXVRRKSIKd(ErbB4-pY664)
FGTVXKGIWVPEd(ErbB4-pY733)
CLLEXVHEHKDNd(ErbB4-pY807)
KGMMXLEERRLVd(ErbB4-pY833)
DEKEXNADGGKMd(ErbB4-pY875)
ECIHXRKFTHQSd(ErbB4-pY894*NS)
DVWSXGVTIWELd(ErbB4-pY906)
GGKPXDGIPTREd(ErbB4-pY921*3)
TIDVXMVMVKCWd(ErbB4-pY950*7NS)
DPQRXLVIQGDEd(ErbB4-pY984*5)
DAEEXLVPQAFN (ErbB4-pY1022*6)
PPPIXTSRARIDd(ErbB4-pY1035*NI)
PPPAXTPMSGNQd(ErbB4-pY1056)
NQFVXRDGGFAAd(ErbB4-pY1066*NI)
VSVPXRAPTSTI (ErbB4-pY1081)
STQRXSADPTVFd(ErbB4-pY1128)
DEEGXMTPMRDK (ErbB4-pY1150)
PKQEXLNPVEENd(ErbB4-pY1162)
DNPEXHNASNGPd(ErbB4-pY1188)
AEDEXVNEPLYLd(ErbB4-pY1202)
NEPLXLNTFANTd(ErbB4-pY1208)
GKAEXLKNNILSd(ErbB4-pY1221)
DNPDXWNHSLPPd(ErbB4-pY1242)
QHPDXLQEYSTKd(ErbB4-pY1258)
YLQEXSTKYFYK (ErbB4-pY1262)
YSTKXFYKQNGRd(ErbB4-pY1266*NI)
TKYFXKQNGRIRd(ErbB4-pY1268*NI)
ENPEXLSEFSLKd(ErbB4-pY1284)
PPPPXRHRNTVVd(ErbB4-pY1301*NI)
X, pY phosphotyrosine
*1 - 7 homologous peptides
*NI no positive interaction hits
*NS peptide not synthesized
Neither
FP only
PM only
Both
NI or NS
