# Supplementary material for: Comprehensive Binary Interaction Mapping of SH2 Domains via Fluorescence Polarization Reveals Novel Functional Diversification of ErbB Receptors
Source: PLoS One. 2012 Sep 4;7(9):e44471. doi: 10.1371/journal.pone.0044471 (PMC3433420; doi:10.1371/journal.pone.0044471)
Supplement: Figure S11 — Inclusion of DTT causes dramatic reduction of intensities of spots on protein microarrays following probing with both cysteine and non-cysteine containing phosphopeptides. Shown are blocks of printed SH2 and PTB domains probed with 1 µM concentrations of indicated fluorescent ErbB peptide solutions (sequences indicated with phosphotyrosine designated by a red “X” and the asterisk denoting the N-terminal rhodamine label) containing 0 mM (-DTT, left panels) or 5 mM DTT (+DTT, right panels). Red spots (upper panels) indicate printed SH2 domains which were printed with trace amounts of Cy5-labeled bovine serum albumin for spot finding purposes during microarray analysis. Rhodamine-labeled peptides bound to arrayed SH2 domains are green in color (lower panels). Spot identities for several SH2 domains are indicated for interactions observed probing with the peptide derived from ErbB2-pY1139 (upper left quadrant of images). (PDF) [file pone.0044471.s011.pdf]

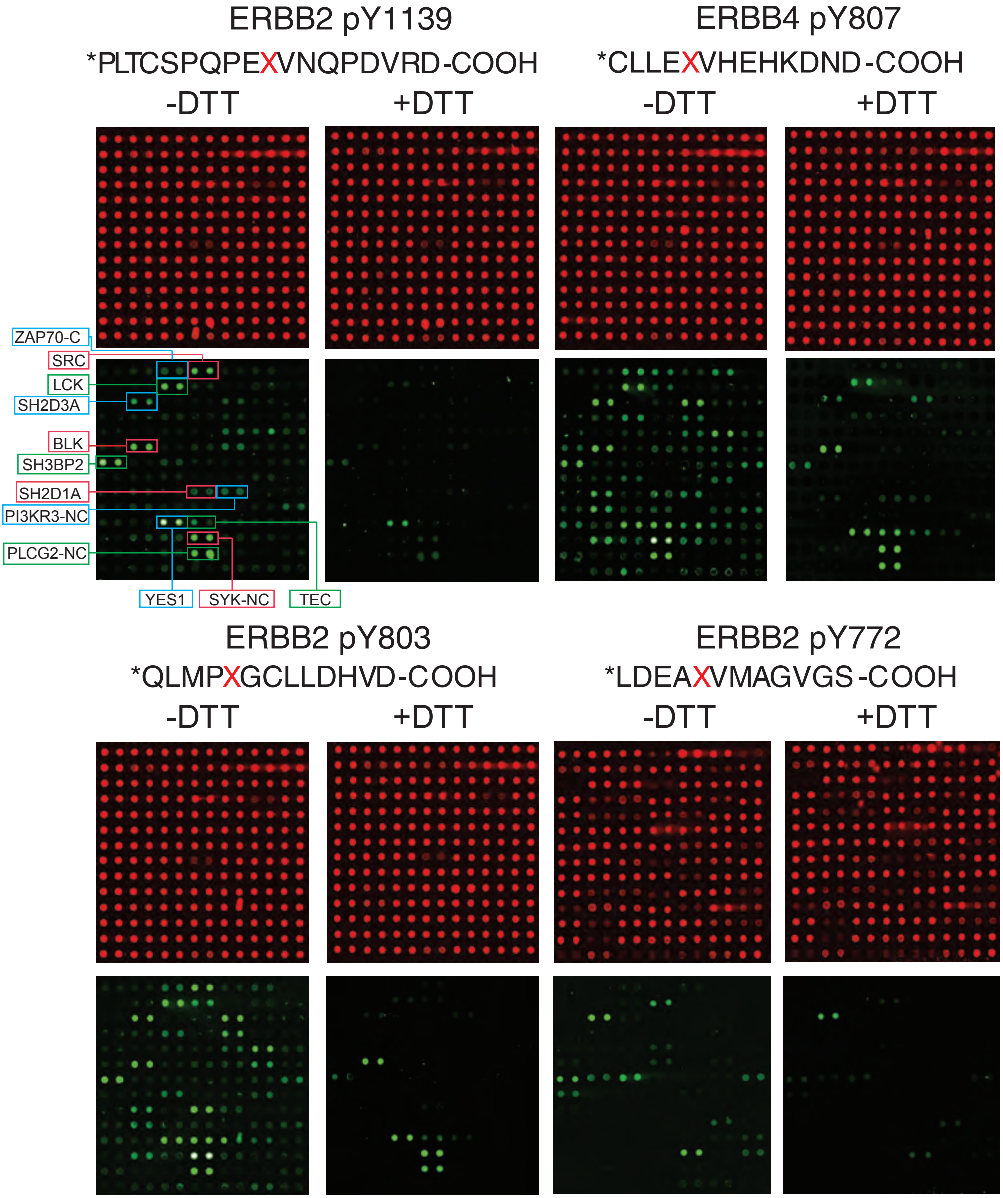

ERBB2 pY1139
*PLTCSPQPEXVNQPDVRD-COOH
-DTT
+DTT
ERBB4 pY807
*CLLEXVHEHKDND-COOH
-DTT
+DTT
ZAP70-C
SRC
LCK
SH2D3A
BLK
SH3BP2
SH2D1A
PI3KR3-NC
PLCG2-NC
YES1
SYK-NC
TEC
ERBB2 pY803
*QLMPXGCLLDHVD-COOH
-DTT
+DTT
ERBB2 pY772
*LDEAXVMAGVGS-COOH
-DTT
+DTT
