# Supplementary material for: Comprehensive Binary Interaction Mapping of SH2 Domains via Fluorescence Polarization Reveals Novel Functional Diversification of ErbB Receptors
Source: PLoS One. 2012 Sep 4;7(9):e44471. doi: 10.1371/journal.pone.0044471 (PMC3433420; doi:10.1371/journal.pone.0044471)
Supplement: Figure S16 — FP determined SH2 domain recruitment activity by ErbB receptors as a product of free energy potential. Color-coded heat maps (see legend) depict free energy contributions for FP interactions between SH2/PTB domains and phosphopeptides representing all potential ErbB1, ErbB2, ErbB3, and ErbB4 phosphotyrosine sites. Binding strength is color coded as indicated and represents free energy as a percentage of total recruitment potential of an SH2/PTB across all ErbB peptides for a particular receptor. Homologous ErbB peptides with identical amino acid residues from +1 to the +4 position relative to the phosphotyrosine (X) are indicated with an asterisk followed by the number (in order of occurrence) of the homologous receptor. Sequences of peptides used are indicated for each homologous receptor site. Lower-case “d”denotes the aspartic acid (Asp) residue pre-charged on the peptide synthesis resin and not a naturally occurring Asp. Peptides that resulted in no positive interactions are designated “NI”; peptides that were unable to be synthesized are designated “NS”. Rows of the heatmaps for these peptides are grayed out to indicate that our FP assay could neither confirm nor deny positive or negative interactions for these peptides. (PDF) [file pone.0044471.s016.pdf]

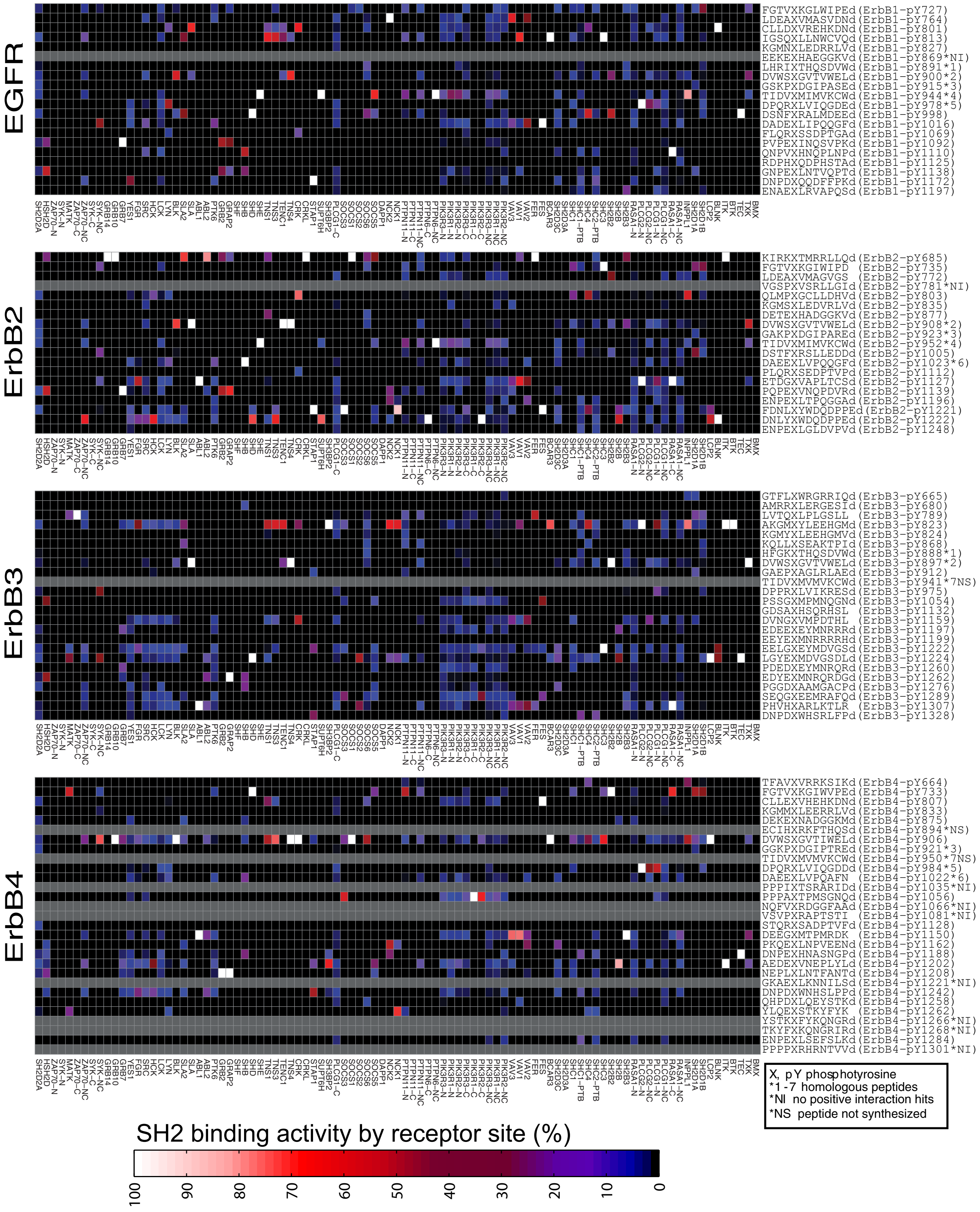
EGFR
FGTVXKGLWIPEd(ErbB1-pY727)
LDEAXVMASVDNd(ErbB1-pY764)
CLLDXVREHKDNd(ErbB1-pY801)
IGSQXLLNWCVQd(ErbB1-pY813)
KGMNXLEDRRLVd(ErbB1-pY827)
EEKEXHAEGGKVd(ErbB1-pY869*NI)
LHRIXTHQSDVWd(ErbB1-pY891*1)
DVWSXGVTVWELd(ErbB1-pY900*2)
GSKPXDGIPASEd(ErbB1-pY915*3)
TIDVXMIMVKCWd(ErbB1-pY944*4)
DPQRXLVIQGDEd(ErbB1-pY978*5)
DSNFXRALMDEEd(ErbB1-pY998)
DADEXLIPQQGFd(ErbB1-pY1016)
FLQRXSSDPTGAd(ErbB1-pY1069)
PVPEXINQSVPKd(ErbB1-pY1092)
QNPVXHNQPLNPd(ErbB1-pY1110)
RDPHXQDPHSTAd(ErbB1-pY1125)
GNPEXLNTVQPTd(ErbB1-pY1138)
DNPDXQQDFFPKd(ErbB1-pY1172)
ENAEXLRVAPQSd(ErbB1-pY1197)
SH2D2A HSH2D ZAP70-N SYK-N MATK ZAP70-C SYK-C ZAP70-NC SYK-NC GRB14 GRB10 GRB7 YES1 FGR SRC HCK LCK LYN BLK SLA2 SLA ABL1 ABL2 PTK6 GRB2 GRAP2 SHF SHB SHD SHE TNS1 TNS3 TENC1 TNS4 CRK CRKL STAP1 SH3BP2 SUPT6H PLCG1-C SOCS3 SOCS1 SOCS2 SOCS6 SOCS5 DAPP1 NCK1 NCK2 PTPN11-N PTPN11-C PTPN11-NC PTPN6-C PTPN6-NC PIK3R3-N PIK3R1-N PIK3R2-N PIK3R3-C PIK3R1-C PIK3R2-C PIK3R3-NC PIK3R1-NC PIK3R2-NC VAV3 VAV1 VAV2 FER FES BCAR3 SH2D3A SH2D3C SHC1 SHC1-PTB SHC4 SHC2-PTB SHC3 SH2B2 SH2B SH2B3 RASA1-N PLCG2-N PLCG2-NC PLCG1-N PLCG1-NC RASA1-C RASA1-NC INPPL1 SH2D1A SH2D1B LCP2 BLNK ITK BTK TEC TXK BMX
ErbB2
KIRKXTMRRLLQd(ErbB2-pY685)
FGTVXKGIWIPD (ErbB2-pY735)
LDEAXVMAGVGS (ErbB2-pY772)
VGSPXVSRLLGId(ErbB2-pY781*NI)
QLMPXGCLLDHVd(ErbB2-pY803)
KGMSXLEDVRLVd(ErbB2-pY835)
DETEXHADGGKVd(ErbB2-pY877)
DVWSXGVTVWELd(ErbB2-pY908*2)
GAKPXDGIPAREd(ErbB2-pY923*3)
TIDVXMIMVKCWd(ErbB2-pY952*4)
DSTFXRSLLEDDd(ErbB2-pY1005)
DAEEXLVPQQGFd(ErbB2-pY1023*6)
PLQRXSEDPTVPd(ErbB2-pY1112)
ETDGXVAPLTCSd(ErbB2-pY1127)
PQPEXVNQPDVRd(ErbB2-pY1139)
ENPEXLTPQGGAd(ErbB2-pY1196)
FDNLXYWDQDPPEd(ErbB2-pY1221)
DNLYXWDQDPPEd(ErbB2-pY1222)
ENPEXLGLDVPVd(ErbB2-pY1248)
ErbB3
GTFLXWRGRRIQd(ErbB3-pY665)
AMRRXLERGESId(ErbB3-pY680)
LVTQXLPLGSLL (ErbB3-pY789)
AKGMXYLEEHGMd(ErbB3-pY823)
KGMYXLEEHGMVd(ErbB3-pY824)
KQLLXSEAKTPId(ErbB3-pY868)
HFGKXTHQSDVWd(ErbB3-pY888*1)
DVWSXGVTVWELd(ErbB3-pY897*2)
GAEPXAGLRLAEd(ErbB3-pY912)
TIDVXMVMVKCWd(ErbB3-pY941*7NS)
DPPRXLVIKRESd(ErbB3-pY975)
PSSGXMPMNQGNd(ErbB3-pY1054)
GDSAXHSQRHSL (ErbB3-pY1132)
DVNGXVMPDTHL (ErbB3-pY1159)
EDEEXEYMNRRRd(ErbB3-pY1197)
EEYEXMNRRRRHd(ErbB3-pY1199)
EELGXEYMDVGSd(ErbB3-pY1222)
LGYEXMDVGSDLd(ErbB3-pY1224)
PDEDXEYMNRQRd(ErbB3-pY1260)
EDYEXMNRQRDGd(ErbB3-pY1262)
PGGDXAAMGACPd(ErbB3-pY1276)
SEQGXEEMRAFQd(ErbB3-pY1289)
PHVHXARLKTLR (ErbB3-pY1307)
DNPDXWHSRLFPd(ErbB3-pY1328)
ErbB4
TFAVXVRRKSIKd(ErbB4-pY664)
FGTVXKGIWVPEd(ErbB4-pY733)
CLLEXVHEHKDNd(ErbB4-pY807)
KGMMXLEERRLVd(ErbB4-pY833)
DEKEXNADGGKMd(ErbB4-pY875)
ECIHXRKFTHQSd(ErbB4-pY894*NS)
DVWSXGVTIWELd(ErbB4-pY906)
GGKPXDGIPTREd(ErbB4-pY921*3)
TIDVXMVMVKCWd(ErbB4-pY950*7NS)
DPQRXLVIQGDDd(ErbB4-pY984*5)
DAEEXLVPQAFN (ErbB4-pY1022*6)
PPPIXTSRARIDd(ErbB4-pY1035*NI)
PPPAXTPMSGNQd(ErbB4-pY1056)
NQFVXRDGGFAAd(ErbB4-pY1066*NI)
VSVPXRAPTSTI (ErbB4-pY1081*NI)
STQRXSADPTVFd(ErbB4-pY1128)
DEEGXMTPMRDK (ErbB4-pY1150)
PKQEXLNPVEENd(ErbB4-pY1162)
DNPEXHNASNGPd(ErbB4-pY1188)
AEDEXVNEPLYLd(ErbB4-pY1202)
NEPLXLNTFANTd(ErbB4-pY1208)
GKAEXLKNNILSd(ErbB4-pY1221*NI)
DNPDXWNHSLPPd(ErbB4-pY1242)
QHPDXLQEYSTKd(ErbB4-pY1258)
YLQEXSTKYFYK (ErbB4-pY1262)
YSTKXFYKQNGRd(ErbB4-pY1266*NI)
TKYFXKQNGRIRd(ErbB4-pY1268*NI)
ENPPXLSEFSLKd(ErbB4-pY1284)
PPPPXRHRNTVVd(ErbB4-pY1301*NI)
X, pY phosphotyrosine
*1-7 homologous peptides
*NI no positive interaction hits
*NS peptide not synthesized
SH2 binding activity by receptor site (%)
100 90 80 70 60 50 40 30 20 10 0
