# Supplementary material for: Comprehensive Binary Interaction Mapping of SH2 Domains via Fluorescence Polarization Reveals Novel Functional Diversification of ErbB Receptors
Source: PLoS One. 2012 Sep 4;7(9):e44471. doi: 10.1371/journal.pone.0044471 (PMC3433420; doi:10.1371/journal.pone.0044471)
Supplement: Methods SI — A detailed description of all biochemical and mathematical analysis methods used in this study. (DOCX) [file pone.0044471.s034.docx]

Supplementary Methods

**SH2 and PTB domain proteins**

The selected 93 SH2 and 2 PTB plasmid constructs and the corresponding vector control were expressed and purified as previously described [1] with the following protocol modifications: 1) BL21(DE3)pLysS *E. coli* were transformed and grown in LB agar and broth, respectively, supplemented with both 100 µg/mL ampicillin and 50 µg/mL chloramphenicol; 2) Isopropyl-1-thio-β-D-galactopyranoside (IPTG, Gold Biotechnology, Inc., St. Louis, MO) was added to cultures at a final concentration of 1 mM for 1-2 hours at 37^o^C; 3) 1mM PMSF, 1 mM benzamidine, and 250 U of benzonase (Novagen, Gibbstown, NJ) were added to the lysis buffer followed by a ten minute incubation period at room temperature; 4) cells were sonicated six times on ice for ten seconds with ten second delay intervals; 5) purified proteins were exchanged into freezing buffer (300 mM NaCl, 50 mM Na_2_PO_4_, pH 8, 20% glycerol v/v) via NAP-10 columns (GE Healthcare, Piscataway, NJ). Purified proteins were assessed for appropriate molecular weights and integrity via PhastGel^TM^ electrophoresis on 8-25% gradient gels (GE Healthcare) and subsequent coomassie GelCode Blue staining (Pierce Chemical Co., Rockford, IL) (Figure S1).

**Peptide purification**

Peptide chains were cleaved from source resin using Reagent K [82.5% TFA (v/v), 5% phenol (v/v), 5% water (v/v), 5% thioanisole (v/v), 2.5% 1,2-ethanedithiol (v/v)] and precipitated in diethylether prechilled to -70ºC. Peptides were analyzed and subsequently purified on an Eclipse DXB-C18 preparative column using an Agilent 1200 Series analytical to preparative liquid chromatography system in combination with a 6100 series single quadrupole mass spectrometer (Agilent Technologies, Santa Clara, CA). The LC/MS system was triggered to collect only when the appropriate mass was observed simultaneously with an abundant absorbance signal at 540 nm (the absorbance maxima of rhodamine). 5 mM DTT was added to all cysteine-containing peptides prior to peptide analysis and purification. Solvent was removed by lyophilization and purified peptides were stored as dry powder at -80 °C prior to use. Peptides were dissolved in buffer B (20 mM HEPES, 100 mM KCl, 0.1% Tween-20, 5 mM DTT, pH 7.8) and the concentrations adjusted by measurement of A540. Control, dephosphorylated peptides were generated using calf alkaline phosphatase (CIP, New England Biolabs, Ipswich, MA); 1 U/µg of CIP was incubated with peptides for one hour at 37^o^C according to the manufacturer’s suggested protocol for proteins. Dephosphorylation of peptides was verified by mass spectrometry as indicated by a loss of 80 daltons.

**FP competitor peptide synthesis**

Unlabeled peptides were synthesized using the same methods as their rhodamine labeled counterparts with several exceptions. Firstly, amino acids were activated in situ with one equivalent of 2-(6-Chloro-1H-benzotriazole-1-yl)-1,1,3,3-tetramethylaminium hexafluorophosphate (HCTU), one equivalent of HOBT, and four equivalents of 4-methylmorpholine (NMM) in DMSO. Secondly, amino acids upstream of phosphotyrosine were coupled twice for ten minutes at five-fold molar excess with the exception of leucine, valine, isoleucine, threonine and tryptophan, which were coupled three times for ten minutes. Thirdly, for Fmoc deprotection, a 1:5:44 molar ratio of 1,8-diazabicyclo[5.4.0]undec-7-ene (DBU):piperidine:NMP was used. Phosphotyrosine was pre-activated separately with one equivalent of 2-(7-Aza-1H-benzotriazole-1-yl)-1,1,3,3-tetramethyluronium hexafluorophosphate (HATU) and three equivalents of DIPEA. Amino acids post-phosphotyrosine were coupled as previously described in the methods. Purification protocols were modified to trigger a collection upon both mass detection and detection of abundant absorbance signal at 215 nm.

**Protein microarray analysis**

Microarray slides were scanned at a resolution of 10 µm using a GenePix 4000B microarray scanner and analyzed using GenePix Pro v6.0 software (Molecular Devices, Sunnyvale, CA). To independently visualize protein spots irrespective of peptide binding, samples were printed simultaneously with 400 nM of Cyanine-5 (Cy5) labeled bovine serum albumin (BSA). Arrays were laser scanned simultaneously at 635 nm and 532 nm wavelengths to detect Cy5 and rhodamine signals, respectively. Following spot layout identification via Cy5 labeled BSA, the mean fluorescence intensity of rhodamine for duplicate protein spots was quantified and normalized by subtracting the corresponding mean fluorescence intensity of thioredoxin. Normalized fluorescence values were then imported into MATLAB and K_D_s determined as previously described [1].

The protein microarray false discovery rate ($\frac{FP}{FP+TP}$) (FDR) was calculated by defining false positives (FP) as interactions detected by protein microarrays that were not detected by fluorescence polarization. True positives were interactions detected by both platforms. The protein microarray false negative rate ($\frac{FN}{FN+TP}$) (FNR) was calculated by defining false negatives (FN) as high-confidence interactions (*P* > 0.5) identified by fluorescence polarization that were not detected by protein microarrays.

**Fluorescence polarization (FP) data analysis**

FP was described by the following equation: $\left( P \right)=\frac{I_{parallel}-I_{perpendicular}}{I_{parallel}+I_{perpendicular}}$, where “P” denotes polarization, and “I” denotes the fluorescent intensity relative to the plane of incident light. Experimental values were output as millipolarization (mP) units and imported into MATLAB where the following equation was used to determine dissociation constants (K_D_s) for each protein/peptide pairing by least squares nonlinear regression:

$P_{obs}=\frac{P_{max}\cdot\left[ Protein \right]}{K_{D}+\left[ Protein \right]}$
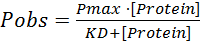
 (1)

Peptides were initially tested for systematic plate effects across protein dilutions and nonspecific binding to thioredoxin by linear regression analysis and any effects were regressed out. The median baseline control polarization value for each plate was then subtracted from each data point to make each binding curve intersect at zero. Plates with excessive variation in baseline control polarizations on both a global and run-specific manner (Dixon’s Q test, p < 0.05) were excluded from further analysis. Outliers in each binding curve were identified and removed using an iterative procedure as follows. The residuals for each fit were used to calculate a global standard deviation of the residuals (GSDR) for each concentration of SH2 domain over all interactions, and individual data points with residuals < or > two GSDR from the global mean residual were flagged as outliers. The outlier with the largest residual from each interaction was removed and nonlinear regression was again performed. This procedure was repeated if additional outliers were identified for an interaction, discarding the interaction if the number of outliers exceeded three. Derived data were then filtered for those interactions that fit well to equation 1 (R^2^ > 0.95), had at least one raw fluorescent polarization value (MaxP) > 15 mP units (indicating sufficient signal over background), and a K_D_ ≤ 20 µM in accordance with previously published filters that optimized concordance with our surface plasmon resonance validation results. Proteins and peptides that failed to yield at least one nominally significant interaction (p ≤ 0.05) in a run given that they did in at least one other run were also excluded from further analysis. Of the 346 peptide-TXN queries performed, for which we do not expect a specific interaction, none have significant binding curves using these criteria. In order to further control our false positive rate against hidden confounding variables producing binding artifacts, we sought to determine whether there was a significant relationship between the strength of an interaction and its technical replication rate at our stringent significance and filtering thresholds. We performed logistic regression on the proportion of successful replicates for each interaction query against K_D_ for all interactions (Figure S15A). Weaker interactions were less likely to replicate than stronger interactions, with a 57.5% chance of replicating a 0.05 K_D_ interaction and a 5.1% chance of replicating a 19.95 K_D_ interaction (p < 0.001, χ^2^ test). This is related to the observation that the majority of interactions detected by FP are tight (Figure S15B). With this logit model, we assigned a probability to each peptide-protein interaction in our data by using a binomial proportions test to compare the observed versus the expected replication rate at the particular K_D_ of that interaction (Table S4). “High-confidence” FP interactions are defined as those having 1 - P(Observed Replication Rate | Expectation) > 0.5 and that have replicated at least twice across independent FP runs. Outlier K_D_ estimates were eliminated (Dixon’s Q test, p < 0.05) and the mean K_D_, standard error (SE), and coefficient of variation (CV) were determined for each interaction. The compiled FP data were organized into heatmaps generated by MATLAB and relative binding strength (µM). Peptides without at least one positive hit were classified as “no interaction” if there was no homologous sequence (i.e. identical at the +1 to +4 positions relative to the phosphotyrosine) that produced a positive interaction within the ErbB receptor family. If there was a homologous peptide, we extrapolated the interaction data to each homologous site. For example, while ErbB4pY1022 was synthesized, it did not produce any interaction hits on our FP assay. However, because it shares homology with ErbB2pY1023, we extrapolated the ErbB2pY1023 interaction date to the homologous site on ErbB4 for later biological inferences (Figure 2).

**Surface plasmon resonance analysis**

A randomly selected set of 12 recombinant SH2 domain proteins and unlabeled (rhodamine dye free) peptides (9 ErbB, 2 GAB1, 2 KIT, 2 MET) were generated for the surface plasmon resonance (SPR) validation study. The 6X His-tagged SH2 domain proteins used in the assay, PLCG1-C, SHC4, VAV2, TNS3, PIK3R3-C, SYK-C, PIK3R1-N, SUPT6H, CRKL, TEC, SHB, and SHC3 were produced and purified as previously described (see “SH2 and PTB domain proteins” section and Jones et al., 2006). Fresh protein stocks were generated so as to avoid bias incurred from proteins previously used in the FP assays. 13-mer peptides corresponding to ErbB1 pY801, ErbB1 pY900, ErbB1 pY978, ErbB1 pY1069, ErbB2 pY685, ErbB pY803, ErbB3 pY912, ErbB4 pY833, ErbB4 pY906, GAB1 pY183, GAB1 pY472, KIT pY570, KIT pY936, MET pY1093 and MET pY1313, were synthesized successfully for the assay using previously described approaches (see “FP competitor peptide synthesis” section). For SPR detection via the Biacore 3000 (GE HealthCare, Piscataway, NJ), His-tagged SH2 domains were immobilized on Biacore NTA sensor chips (GE HealthCare, Piscataway, NJ) pre-charged with Ni^2+^ ions in one flow cell while the second served as a negative control reference cell. Peptides were introduced in solution phase to both cells and relative response units (RUs) were recorded from output sensograms. Nine concentrations of peptide and a buffer control, in duplicate, were used to determine K_D_s using the equilibrium method (best-fit simultaneous modeling for peptide association and disassociation curves).

We leveraged this data to assess the specificity and sensitivity of our high-throughput fluorescence polarization assay. The fluorescence polarization FDR ($\frac{FP}{FP+TP}$) was calculated by defining false positives (FP) as interactions detected by FP that were not detected by SPR. True positives were interactions detected by both platforms. The fluorescence polarization FNR ($\frac{FN}{FN+TP}$) was calculated by defining false negatives (FN) as interactions identified by SPR that were not detected by FP. As expected, the false positive rate (FPR) decreased and the FNR increased as the p-value threshold for accepting an interaction given its replication rate was raised (Figure S8).

***Z’* factor analysis**

Positive and negative controls for 33 peptide-protein interactions were queried on one 384-well plate in technical triplicate and the plate was read five times by a Molecular Devices Analyst GT. We first wanted to ensure that there were no read-specific biases or technical outliers in our fluorescent readings. The average standard deviation of a given interaction’s fluorescence across all reads and technical replicates was 4.86 mP with no significant outliers (Dixon’s Q test, p < 0.05). For each individual peptide-protein query, we then calculated the *Z’* factor using Equation (**2**).

$Z^{'}=1-\frac{(3SD_{bound}+3SD_{free})}{\bar{x}_{bound}-\bar{x}_{free}}$ (2)

The *Z’* factor is used to evaluate the ability of an assay to distinguish between a putative interaction (0% inhibition) and a negative control (no interaction, 100% binding inhibition) by taking into consideration assay signal versus sample and control noise [2].

All analyses were performed in R ([www.R-project.org](http://www.R-project.org)) or MATLAB (The MathWorks, Inc., Natick, MA).

**Comparison to protein-protein interaction databases**

BioGRID (version 3.1.77; [http://thebiogrid.org/download.php](http://www.sciencedirect.com/science?_ob=RedirectURL&_method=externObjLink&_locator=url&_issn=00928674&_origin=article&_zone=art_page&_plusSign=%2B&_targetURL=http%253A%252F%252Fthebiogrid.org%252Fdownload.php))[3]), HPRD (Release_9_041310; [http://www.hprd.org/download](http://www.sciencedirect.com/science?_ob=RedirectURL&_method=externObjLink&_locator=url&_issn=00928674&_origin=article&_zone=art_page&_plusSign=%2B&_targetURL=http%253A%252F%252Fwww.hprd.org%252Fdownload))[4], IntAct ([http://www.ebi.ac.uk/intact/main.xhtml](http://www.sciencedirect.com/science?_ob=RedirectURL&_method=externObjLink&_locator=url&_issn=00928674&_origin=article&_zone=art_page&_plusSign=%2B&_targetURL=http%253A%252F%252Fwww.ebi.ac.uk%252Fintact%252Fmain.xhtml))[5] and iRefWeb (version 3.9, <http://www.wodaklab.org/iRefWeb/>)[6] datasets were downloaded from the above sources. We first prepared these datasets for cross-comparison by selecting all human-to-human binary PPIs and removing self and redundant interactions. We then split our unfiltered FP data into a binary table of unique protein associations where an individual pY site represented that receptor. Table S8 shows the ErbB family interactions identified from the literature-based databases and whether those interactions were identified by FP. The overlap between FP-derived and PM-derived interactions is depicted in Figure S6.

**Protein and gene ontology enrichment analyses**

The total number of ErbB pY sites each SH2-domain containing protein and gene ontology class of proteins bound was first determined. We then performed 10,000 permutations of the pY sites to build a reference distribution of the null hypothesis of each receptor binding a given protein or class of proteins at a random number of sites given the number of sites queried. We defined statistically significant enrichment and depletion of binding sites by identifying instances where the observed number of binding sites was unlikely to occur by chance given the number of sites bound across all receptors (p < 0.05).

References

1. Jones RB, Gordus A, Krall JA, MacBeath G (2006) A quantitative protein interaction network for the ErbB receptors using protein microarrays. Nature 439: 168–174.

2. Zhang, Chung, Oldenburg (1999) A Simple Statistical Parameter for Use in Evaluation and Validation of High Throughput Screening Assays. J Biomol Screen 4: 67–73.

3. Stark C, Breitkreutz B-J, Chatr-aryamontri A, Boucher L, Oughtred R, et al. (2010) The BioGRID Interaction Database: 2011 update. Nucleic Acids Research 39: D698–D704. doi:10.1093/nar/gkq1116.

4. Keshava Prasad TS, Goel R, Kandasamy K, Keerthikumar S, Kumar S, et al. (2009) Human Protein Reference Database--2009 update. Nucleic Acids Research 37: D767–D772. doi:10.1093/nar/gkn892.

5. Aranda B, Achuthan P, Alam-Faruque Y, Armean I, Bridge A, et al. (2009) The IntAct molecular interaction database in 2010. Nucleic Acids Research 38: D525–D531. doi:10.1093/nar/gkp878.

6. Turner B, Razick S, Turinsky AL, Vlasblom J, Crowdy EK, et al. (2010) iRefWeb: interactive analysis of consolidated protein interaction data and their supporting evidence. Database (Oxford) 2010. Available:http://www.ncbi.nlm.nih.gov/pmc/articles/PMC2963317/. Accessed 9 August 2012.
