# Supplementary material for: Comprehensive Binary Interaction Mapping of SH2 Domains via Fluorescence Polarization Reveals Novel Functional Diversification of ErbB Receptors
Source: PLoS One. 2012 Sep 4;7(9):e44471. doi: 10.1371/journal.pone.0044471 (PMC3433420; doi:10.1371/journal.pone.0044471)
Supplement: Figure S10 — 95% confidence intervals for total free binding energy for each receptor for each molecular function. Relative binding free energies of interactions were summed across all domains contained from a particular function. Confidence intervals were calculated by summing ΔΔG +/−1.96 standard deviations derived from the distribution of KD estimates of individual interaction replicates. (PDF) [file pone.0044471.s010.pdf]

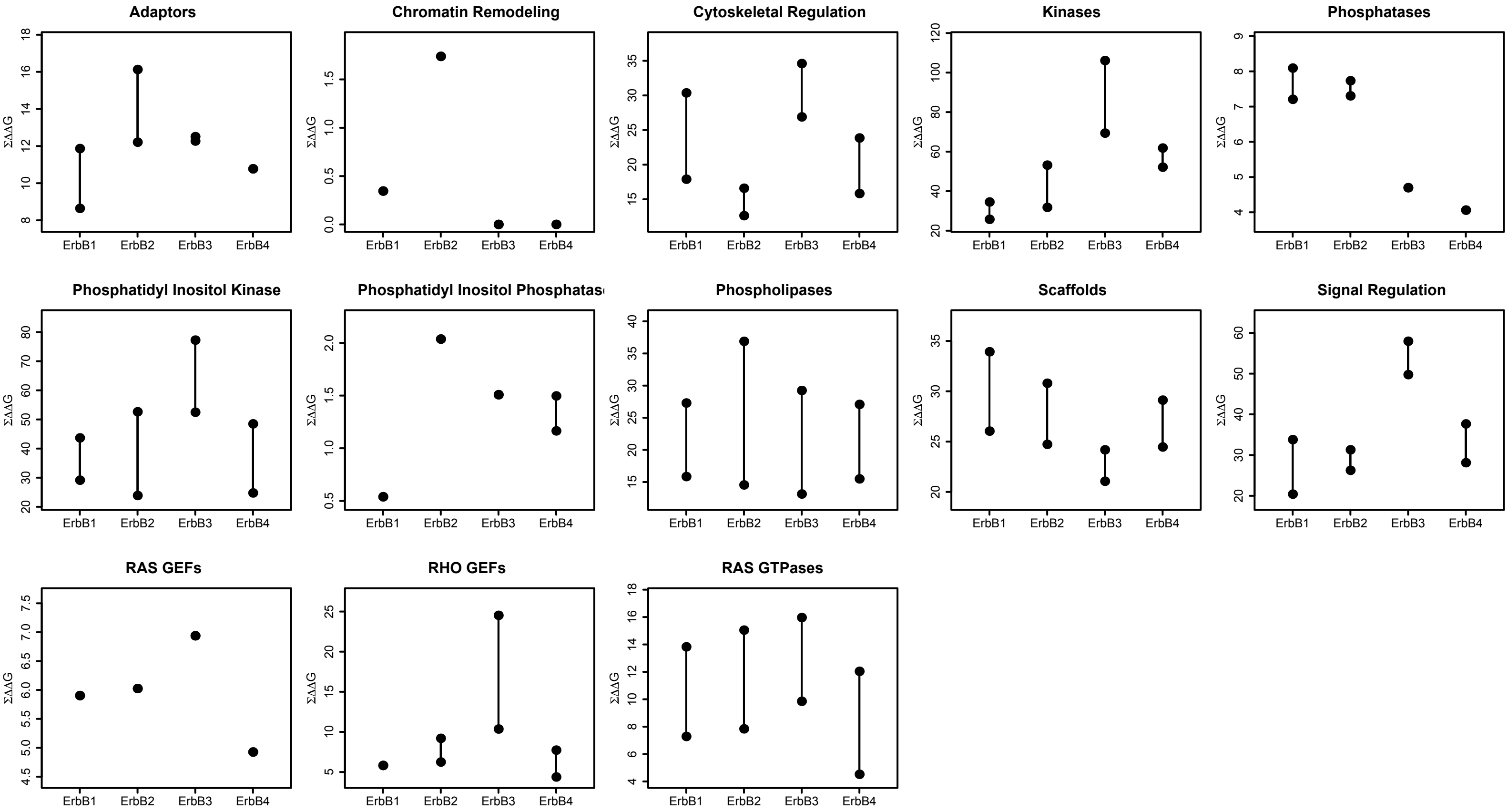
Adaptors
Chromatin Remodeling
Cytoskeletal Regulation
Kinases
Phosphatases
Phosphatidyl Inositol Kinase
Phosphatidyl Inositol Phosphatase
Phospholipases
Scaffolds
Signal Regulation
RAS GEFs
RHO GEFs
RAS GTPases
ΣΔΔG
ErbB1
ErbB2
ErbB3
ErbB4
