# Supplementary material for: Comprehensive Binary Interaction Mapping of SH2 Domains via Fluorescence Polarization Reveals Novel Functional Diversification of ErbB Receptors
Source: PLoS One. 2012 Sep 4;7(9):e44471. doi: 10.1371/journal.pone.0044471 (PMC3433420; doi:10.1371/journal.pone.0044471)
Supplement: Figure S12 — SH2 domain recruitment potential of the ErbB family as determined by protein microarrays in the absence of DTT. Color-coded heat maps (see legend) represent apparent dissociation constants (KDs) for protein microarray interactions between SH2/PTB domains and phosphopeptides representing all potential ErbB1, ErbB2, ErbB3, and ErbB4 phosphotyrosine sites. Binding strength is color coded as indicated. Homologous ErbB peptides with identical amino acid residues from +1 to the +4 position relative to the phosphotyrosine (X) are indicated with an asterisk followed by the number (in order of occurrence) of the homologous receptor. Sequences of peptides used are indicated for each homologous receptor site. Lower-case “d” denotes the aspartic acid (Asp) residue pre-charged on the peptide-synthesis resin and not a naturally-occurring Asp. Peptides that resulted in no positive interactions are designated “NI”; peptides not synthesized for PMs are designated “NS”. Rows of the heatmaps for these peptides are grayed out to indicate that our FP assay could neither confirm nor deny positive or negative interactions from these peptides. (PDF) [file pone.0044471.s012.pdf]

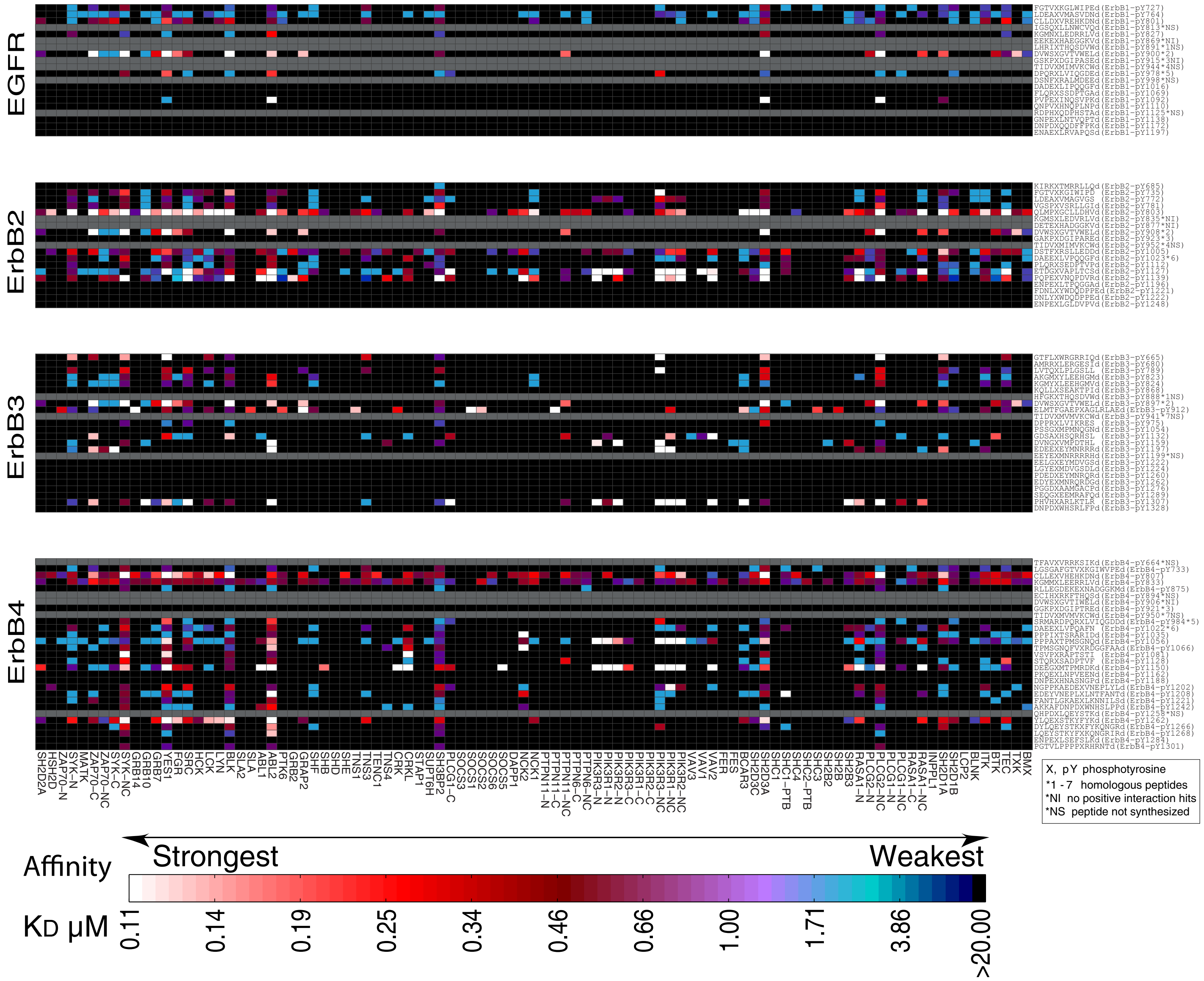
EGFR
ErbB2
ErbB3
ErbB4
X, pY phosphotyrosine
*1 - 7 homologous peptides
*NI no positive interaction hits
*NS peptide not synthesized
Affinity
Strongest
Weakest
KD μM
0.11
0.14
0.19
0.25
0.34
0.46
0.66
1.00
1.71
3.86
>20.00
